# Supplementary material for: Synthesis and Preliminary Biological Evaluation of Two Fluoroolefin Analogs of Largazole Inspired by the Structural Similarity of the Side Chain Unit in Psammaplin A
Source: Mar Drugs. 2019 Jun 3;17(6):333. doi: 10.3390/md17060333 (PMC6628159; doi:10.3390/md17060333)
Supplement: Supplementary file 1 [file marinedrugs-17-00333-s001.pdf]

Supporting Information

Title: Synthesis and preliminary biological evaluation of two fluoroolefin analogs of Largazole inspired by the structural similarity of the side chain unit in Psammaplin A

Authors: Bingbing Zhang [1,†], Guangsheng Shan [1,†], YinYing Zheng [1,†], Xiaoling Yu [1, 2], Zhu-Wei Ruan [1], Yang Li [1] and Xinsheng Lei [1, 3]*

Affiliations:

[1] School of Pharmacy, Fudan University, 826 Zhangheng Road, Pudong Zone, Shanghai 201203, China

[2] College of Chemistry and Molecular Engineering, Zhengzhou University, Zhengzhou, Henan Province 450001, China.

[3] Key Laboratory of Synthetic Chemistry of Natural Substances, Shanghai Institute of Organic Chemistry, Chinese Academy of Sciences, Shanghai 200032, China.

***** Correspondence: leixs@fudan.edu.cn; Tel.: +86-021-51980128

† These authors contributed equally to the work.

Contact email: leixs@fudan.edu.cn

Table of Contents

1. General Experimental S3

2. ^{1}H NMR Spectra of **3** S4

3. ^{1}H, ^{13}C and ^{19}F NMR Spectra of Z-isomer of **4**........ S5-6

4. ^{1}H, ^{13}C and ^{19}F NMR Spectra of E-isomer of **4**........ S7-8

5. ^{1}H, ^{13}C and ^{19}F NMR Spectra of **5**........ S9-10

6. ^{1}H, ^{13}C and ^{19}F NMR Spectra of **8**........ S11-12

7. ^{1}H, ^{13}C and ^{19}F NMR Spectra of **9**........ S13-14

8. ^{1}H, ^{13}C and ^{19}F NMR Spectra of **11**........ S15-16

9. ^{1}H, ^{13}C, ^{19}F NMR, COSY, HMQC and HMBC Spectra of **13a** S17-20

10. ^{1}H, ^{13}C, ^{19}F NMR, COSY, HMQC and HMBC Spectra of **13b** S21-24

11. ^{1}H, ^{13}C and ^{19}F NMR Spectra of **14a** S25-26

12. ^{1}H, ^{13}C and ^{19}F NMR Spectra of **14b** S27-28

13. ^{1}H NMR Spectra of **15a** S29

14. ^{1}H and ^{19}F NMR Spectra of **15b** S30

15. ^{1}H, ^{13}C, ^{19}F NMR, COSY, HMQC and HMBC Spectra of **16a** S31-34

16. ^{1}H, ^{13}C and ^{19}F NMR Spectra of **16b** S35-36

17. The concentration-response curves of the compounds in the enzymatic assays........ S37

18. The concentration-response curves of the compounds in the cellular assays........ S37

Experimental Section

General Methods. The chemicals and reagents were purchased from Acros, Alfa Aesar, and National Chemical Reagent Group Co. Ltd., P. R. China, and used without further purification. Anhydrous solvents (THF, MeOH, DMF, CH_2Cl_2, and CH_3CN) used in the reactions were dried and freshly distilled before use. All the reactions were carried out under Ar atmosphere, otherwise stated else. The progress of the reactions was monitored by TLC (silica-coated glass plates) and visualized under UV light, and by using iodine or phosphomolybdic acid. Melting points were measured on a SGW X-4 microscopy melting point apparatus without correction. ^{1}H NMR and ^{13}C NMR spectra were recorded either on a 400 MHz Varian Instrument at 25 °C or 600 MHz Bruke Instrument at 25 °C, using TMS as an internal standard, respectively. Multiplicity is tabulated as s for singlet, d for doublet, dd for doublet of doublet, t for triplet, and m for multiplet. HRMS spectra were recorded on Finnigan-Mat-95 mass spectrometer, equipped with ESI source.

3-(tritylthio)propanal (3).

^{1}H NMR Spectrum of **3**

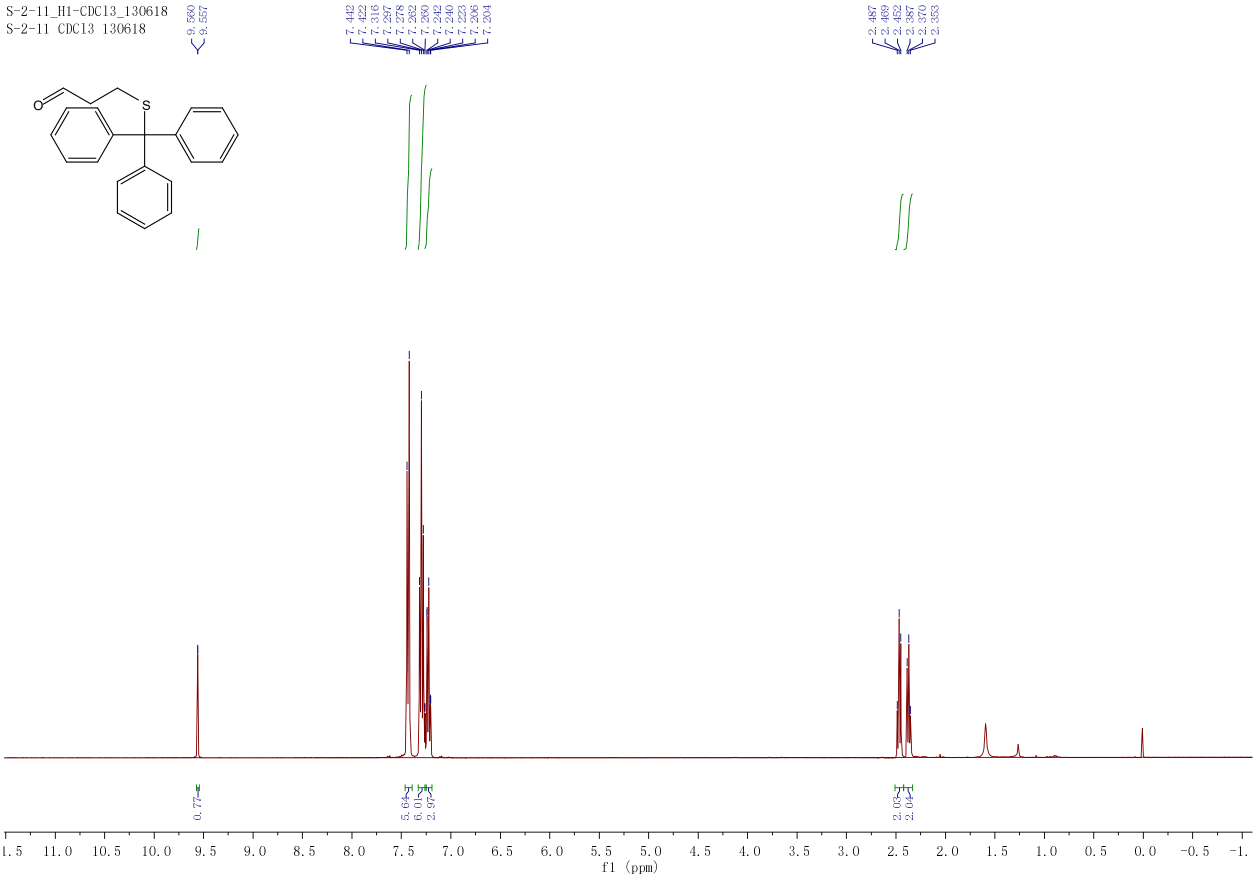

ethyl 2-fluoro-5-(tritylthio)pent-2-enoate (4).

^{1}H NMR Spectrum of **Z-isomer of 4**

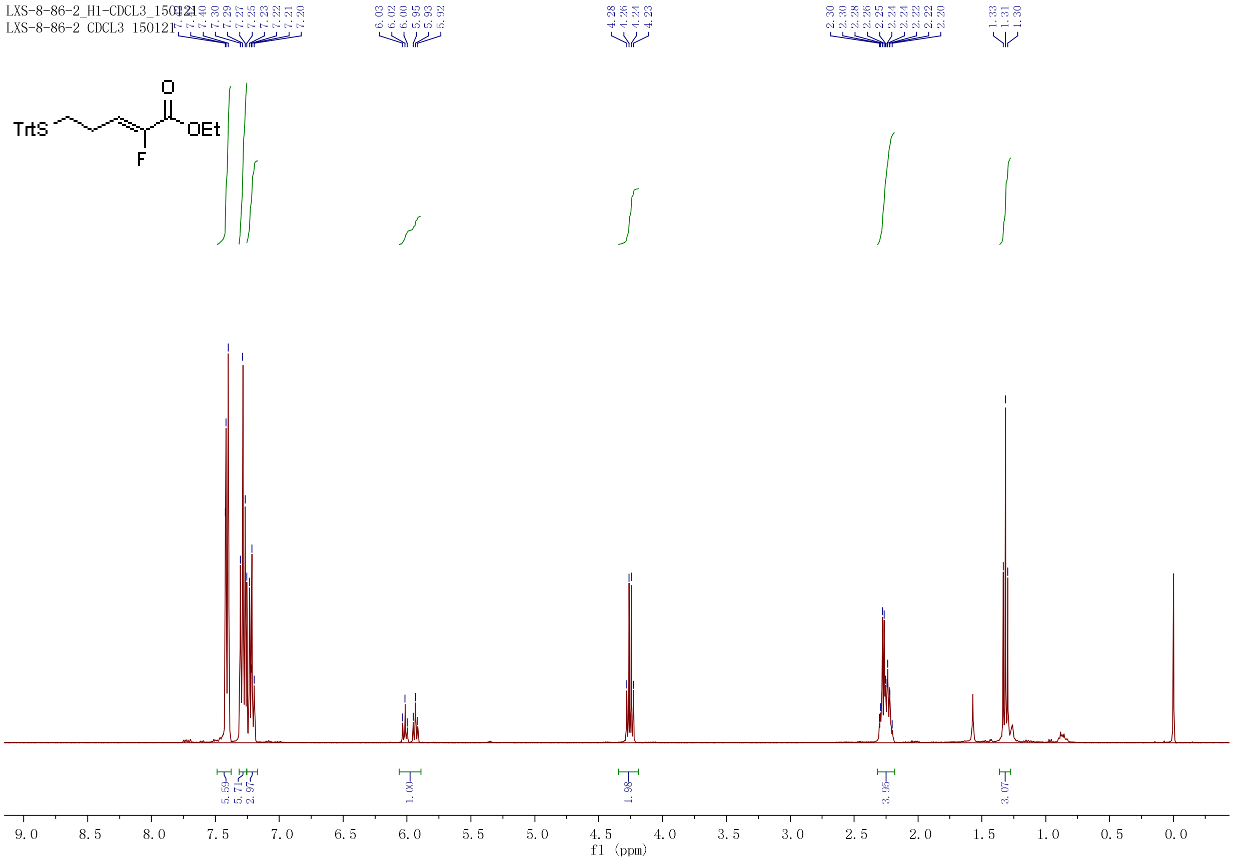

^{13}C NMR Spectrum of **Z-isomer of 4**

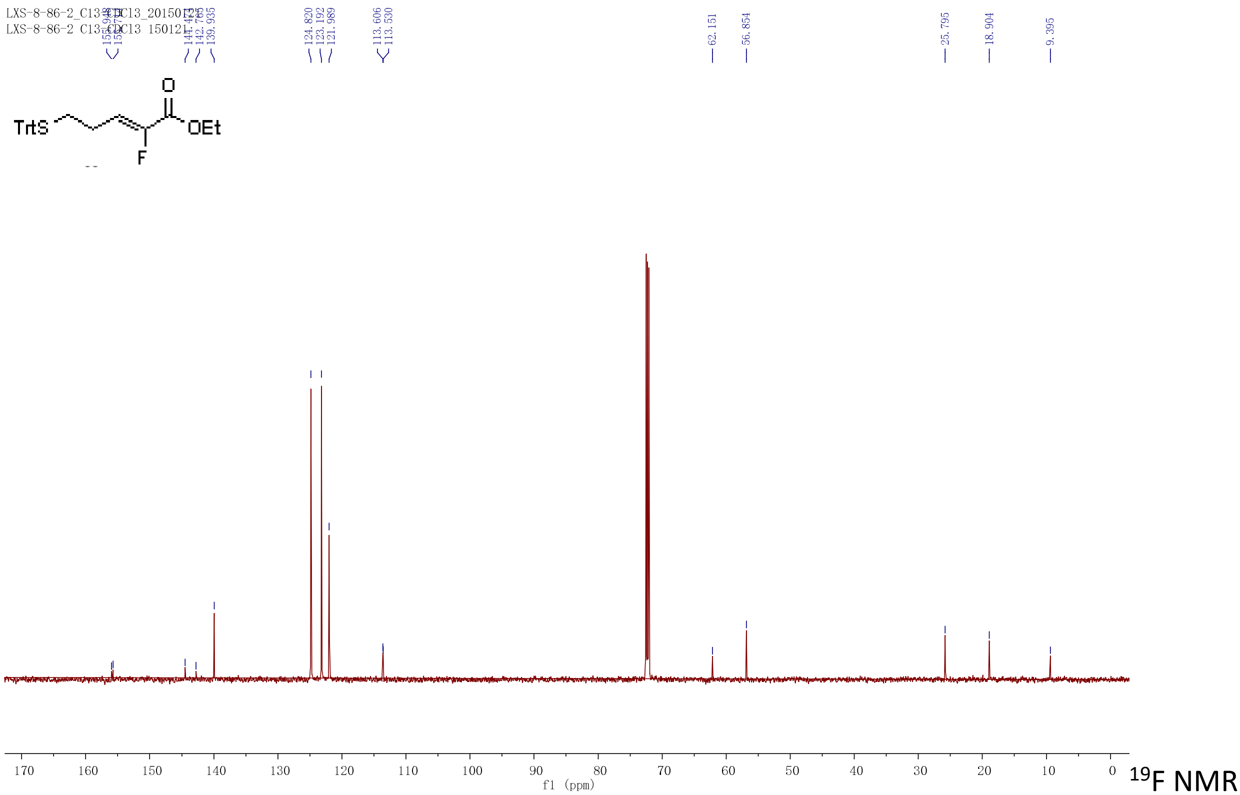

^{19}F NMR Spectrum of **Z-isomer of 4**

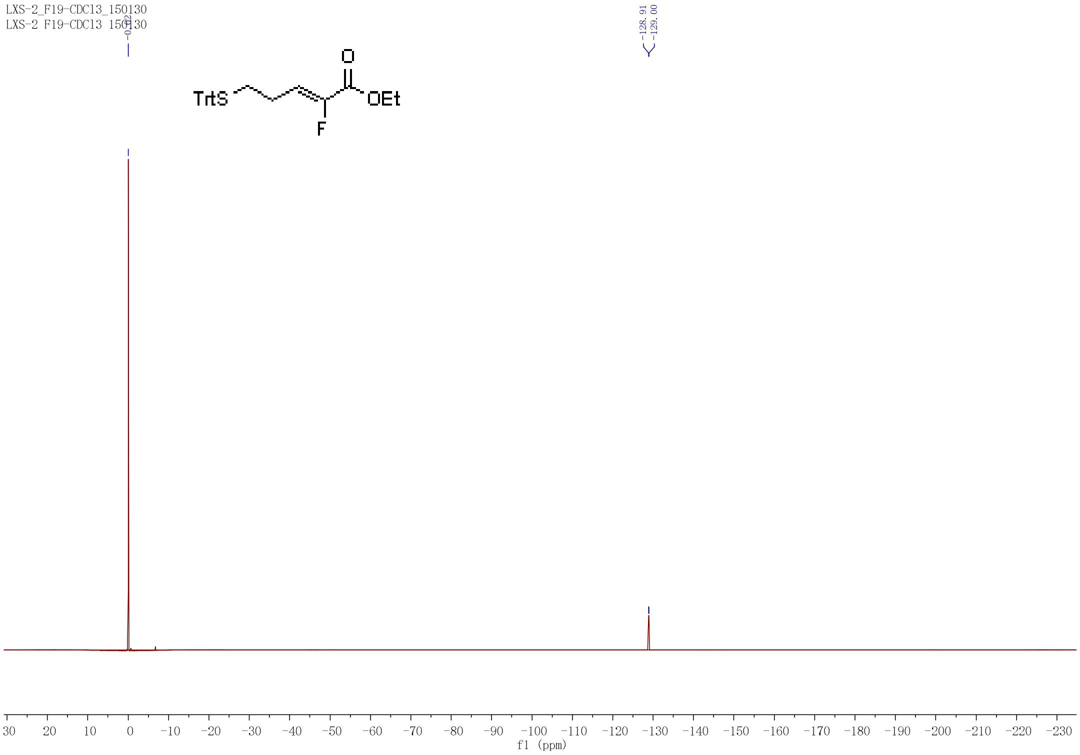

E-isomer of 4

^{1}H NMR Spectrum of **E-isomer of 4**

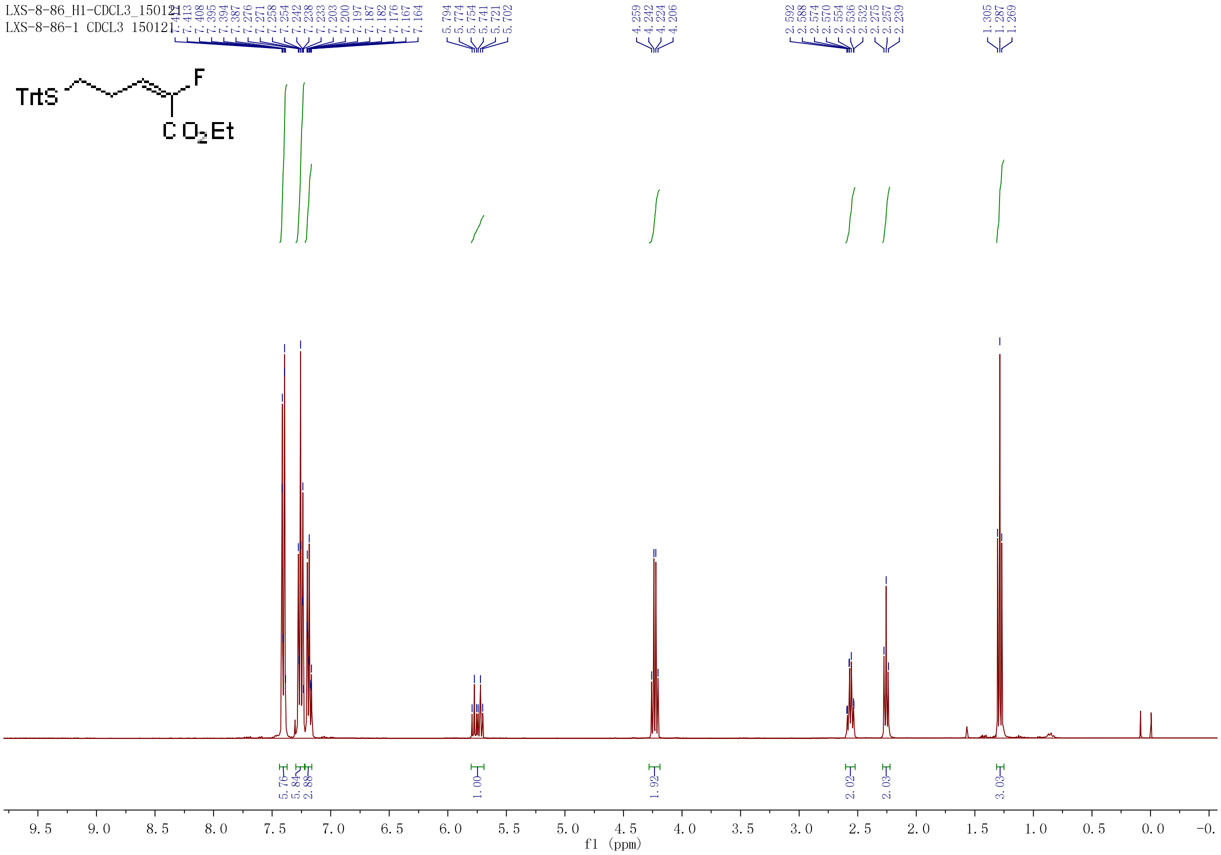

^{13}C NMR Spectrum of **E-isomer of 4**

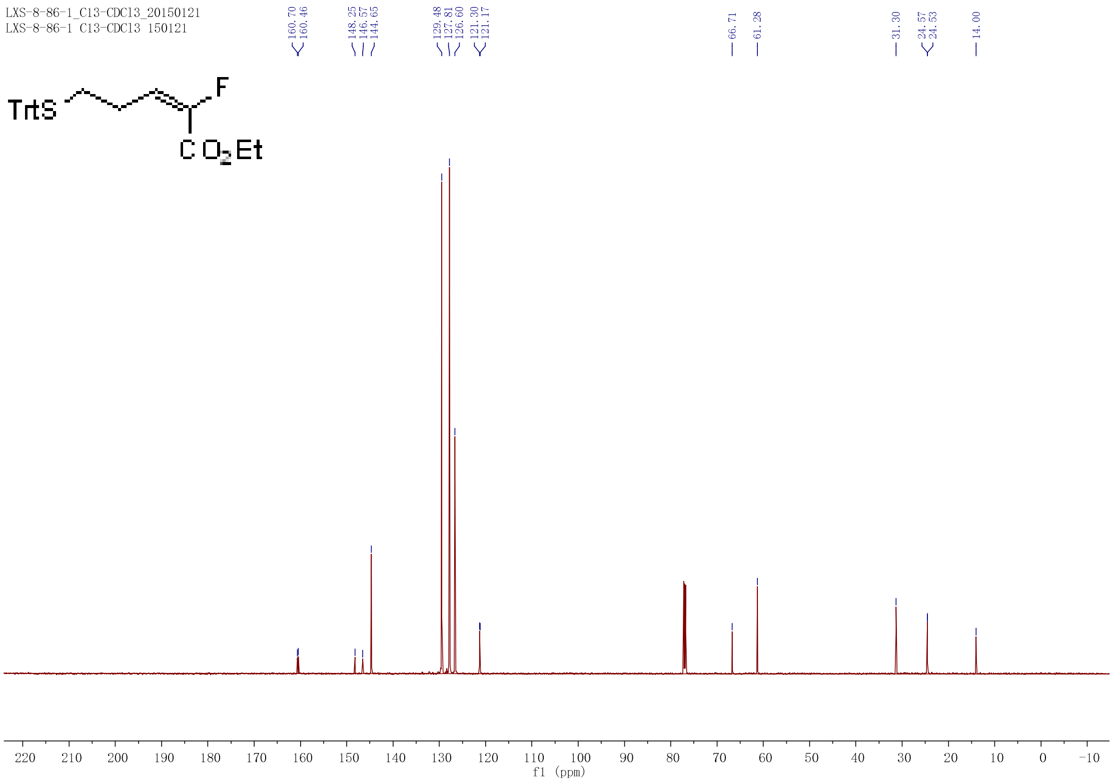

^{19}F NMR Spectrum of **E-isomer of 4**

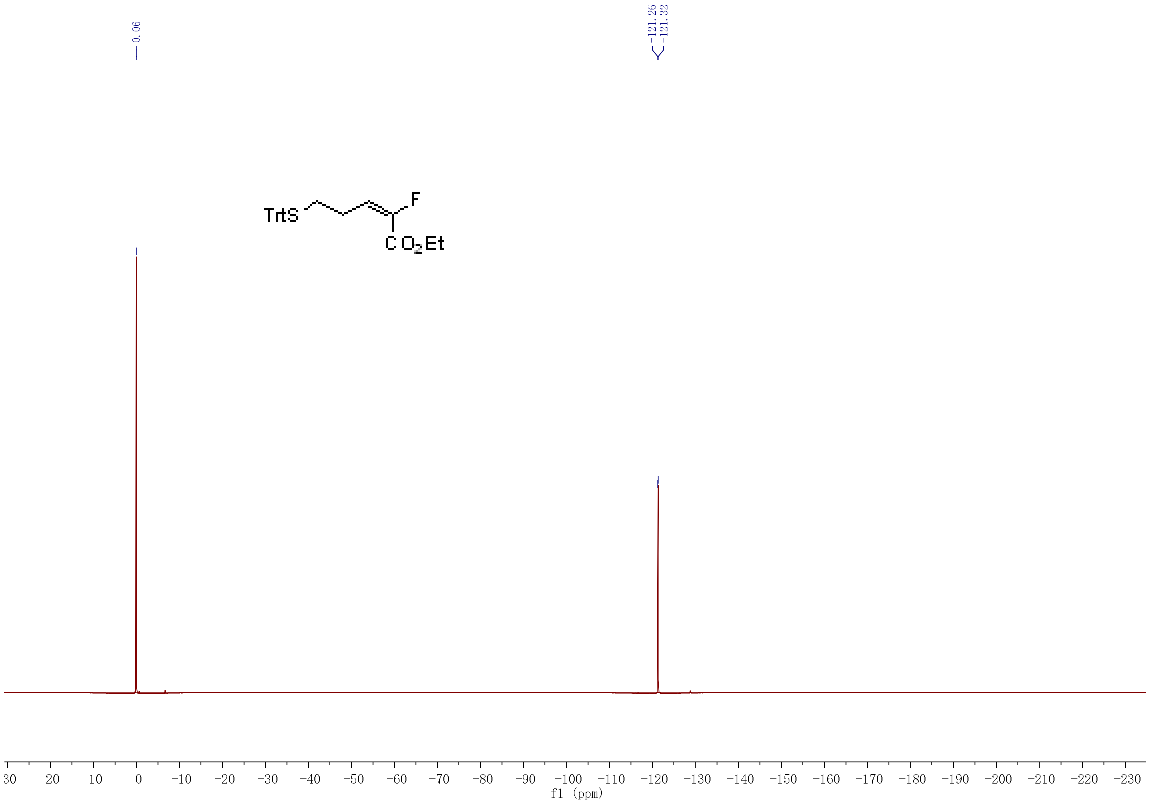

(Z)-2-fluoro-5-(tritylthio)pent-2-enal (5).

^{1}H NMR Spectrum of **5**

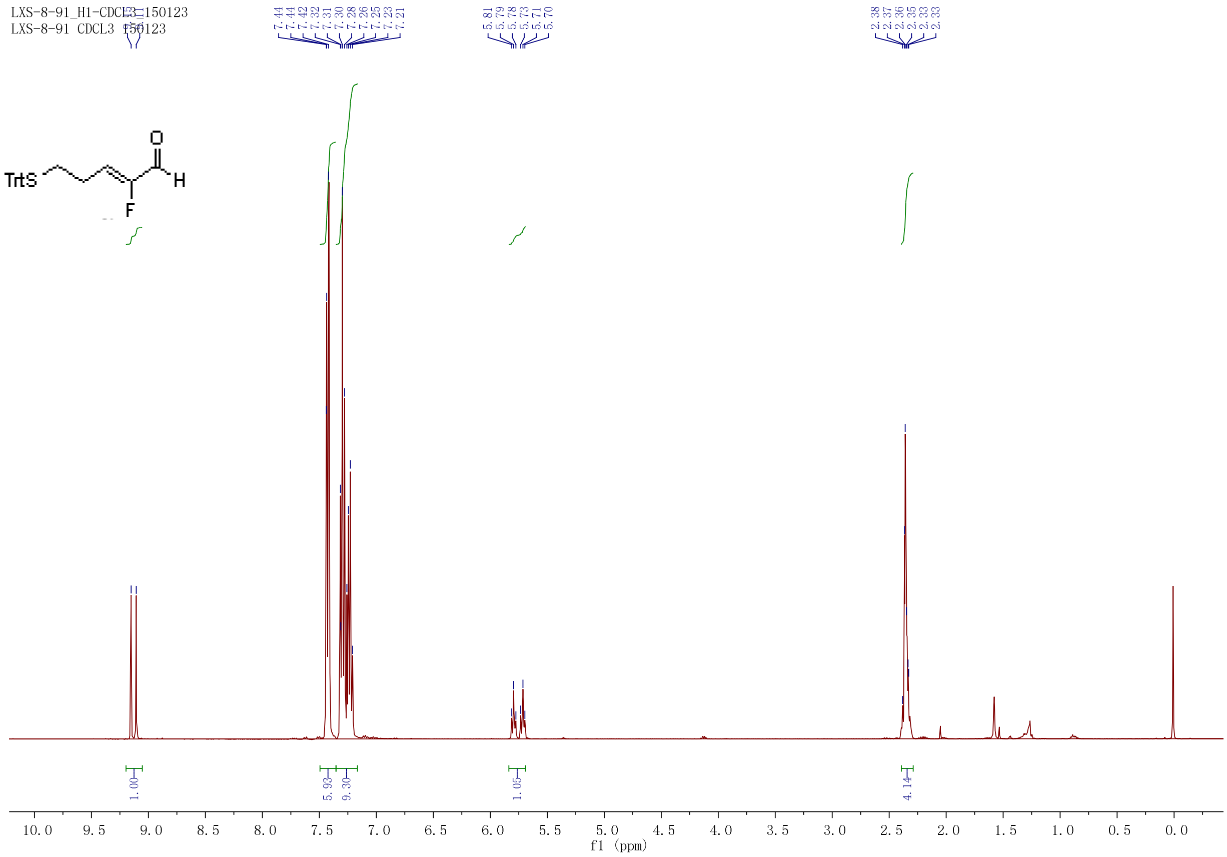

^{13}C NMR Spectrum of **5**

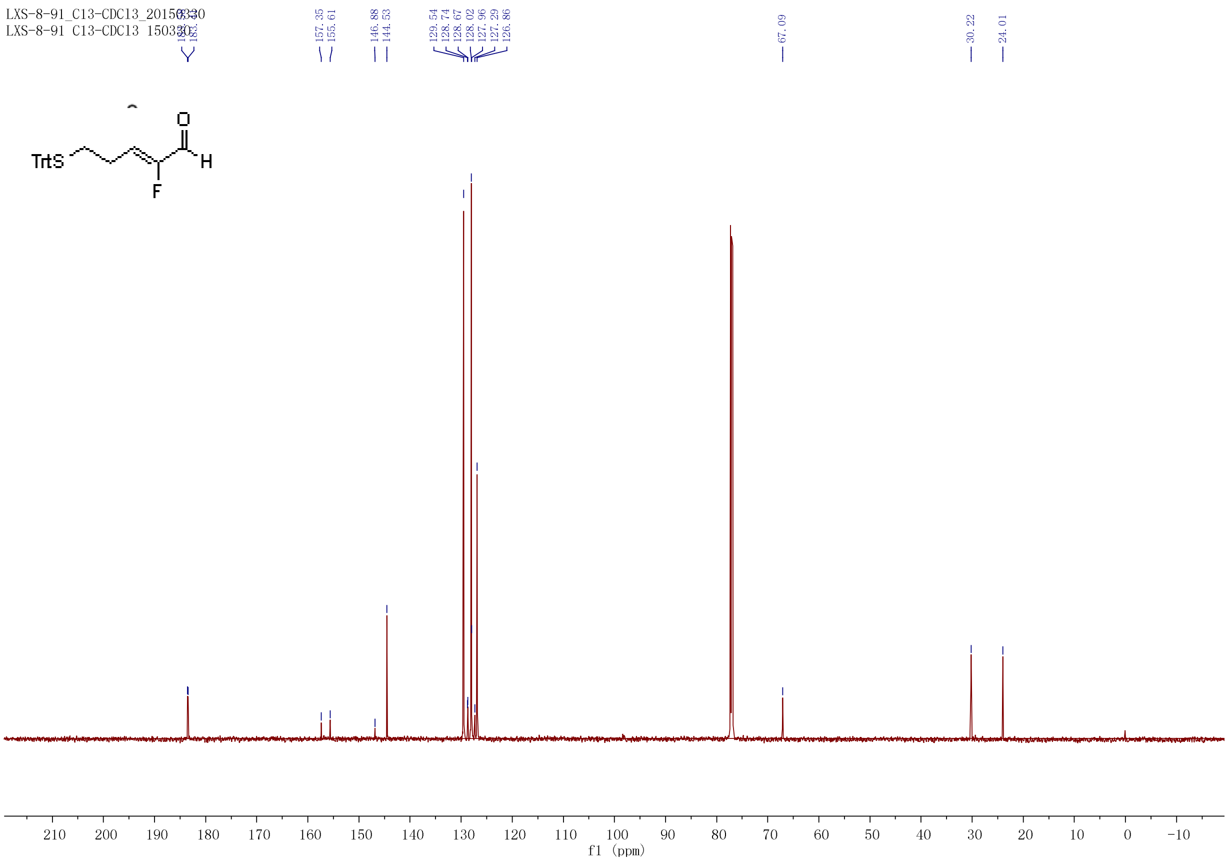

^{19}F NMR Spectrum of **5**

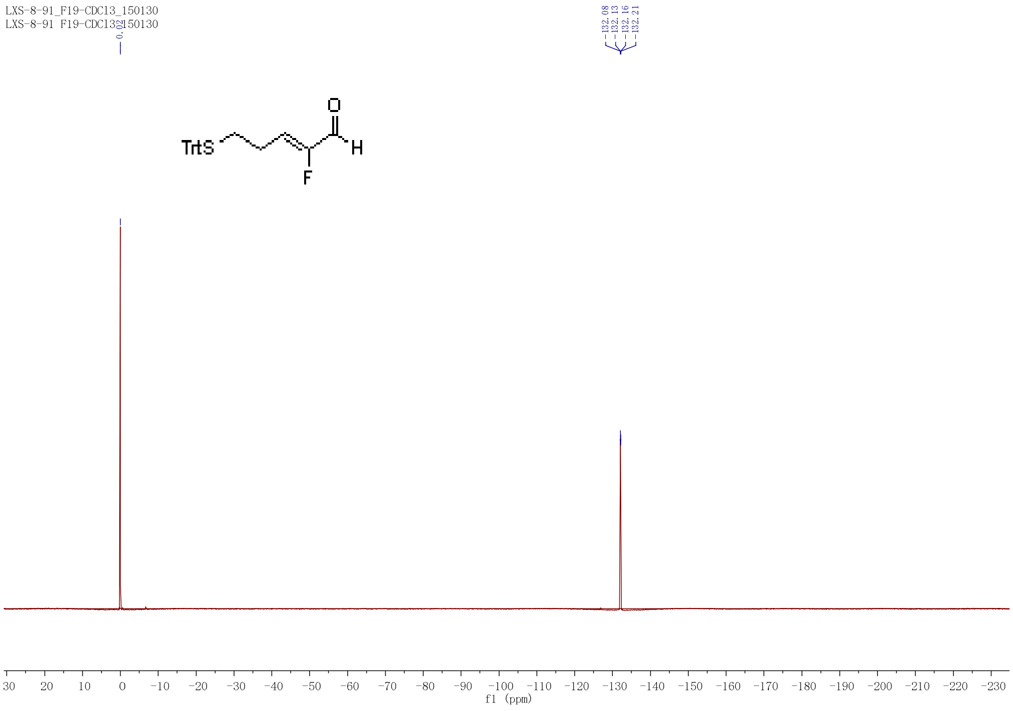

(S,Z)-1-((R)-4-benzyl-2-thioxothiazolidin-3-yl)-4-fluoro-3-hydroxy-7-(tritylthio)hept-4-en-1-one (8).

^{1}H NMR Spectra of **8**

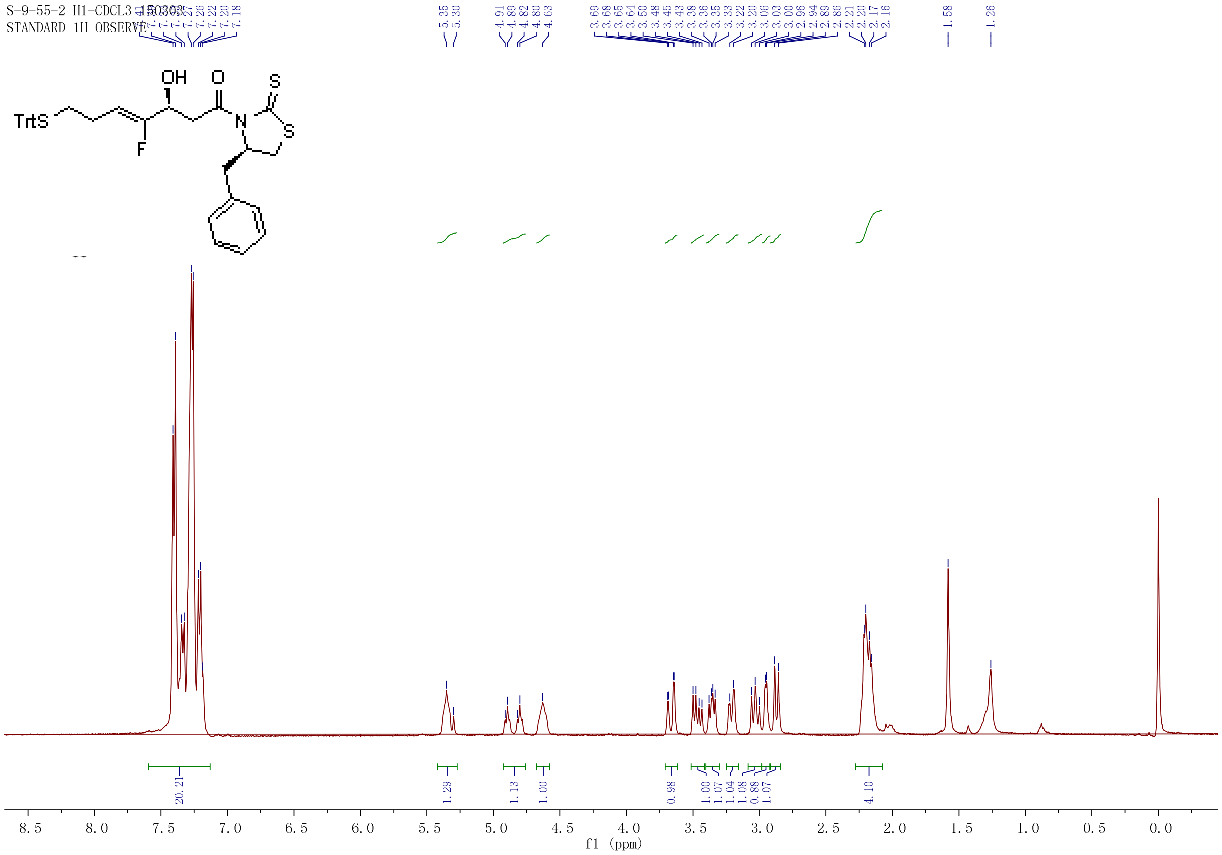

^{13}C NMR Spectra of **8**

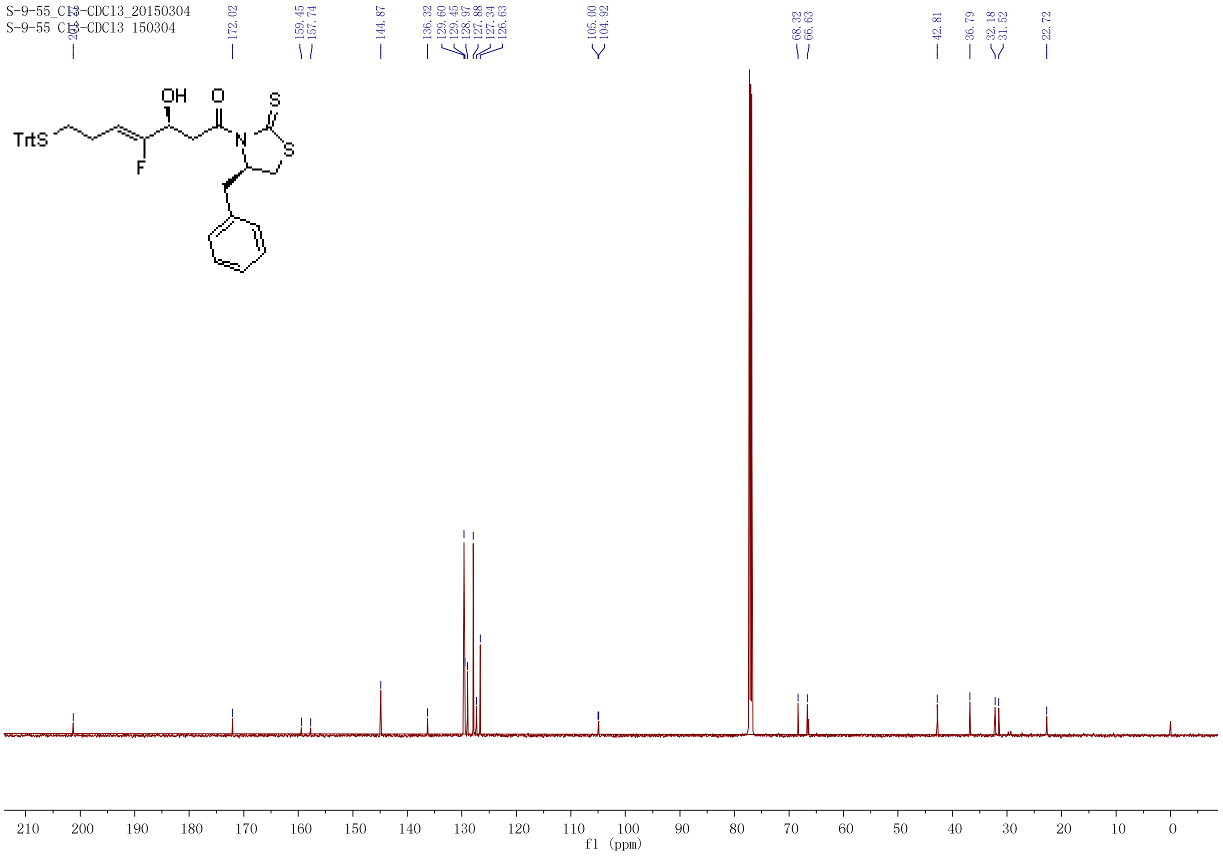

^{19}F NMR Spectra of **8**

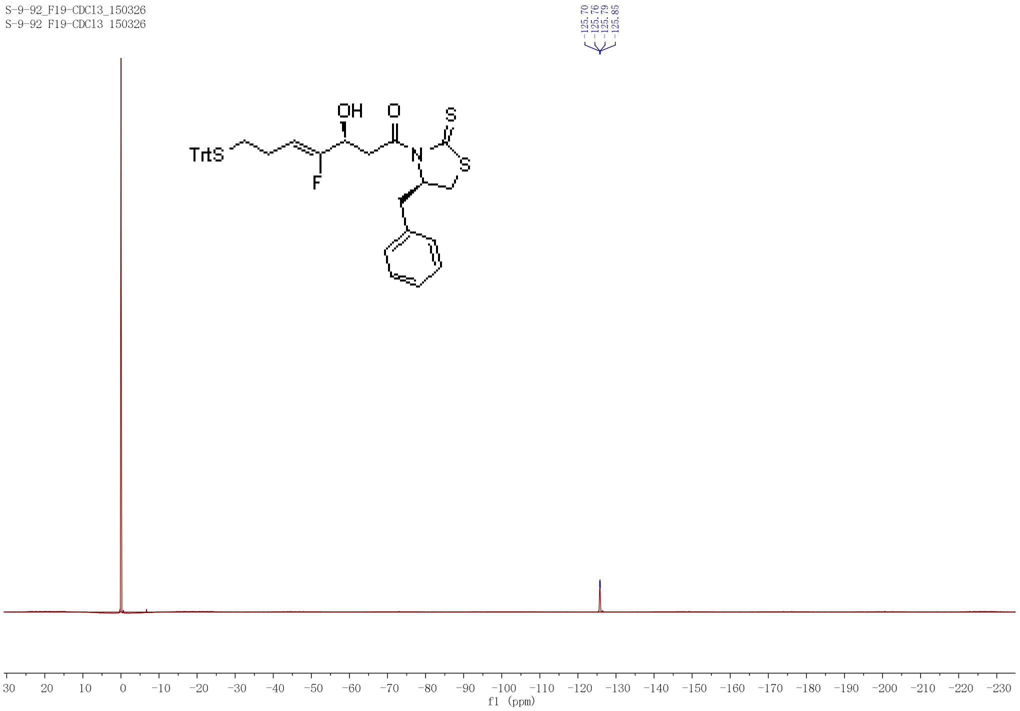

(S,Z)-1-((R)-4-benzyl-2-thioxothiazolidin-3-yl)-4-fluoro-3-hydroxy-7-(tritylthio)hept-4-en-1-one (9).

^{1}H NMR Spectra of **9**

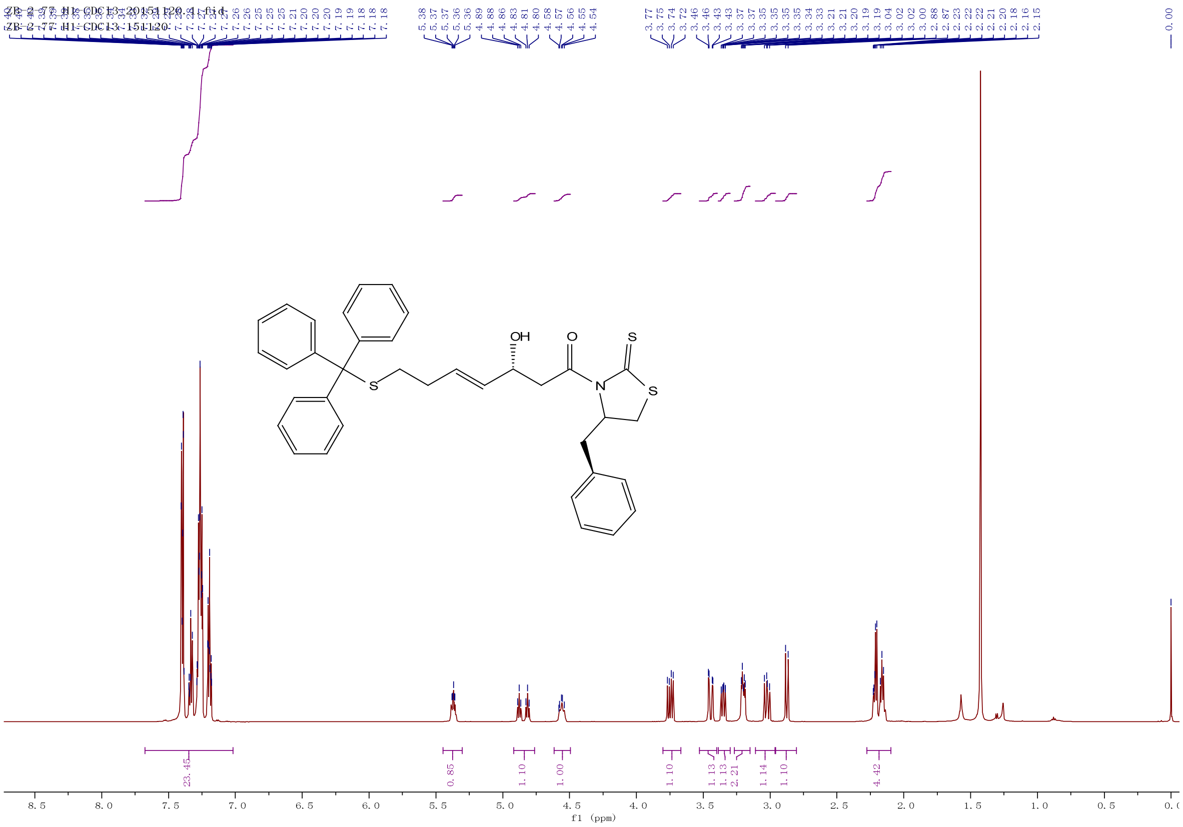

^{13}C NMR Spectra of **9**

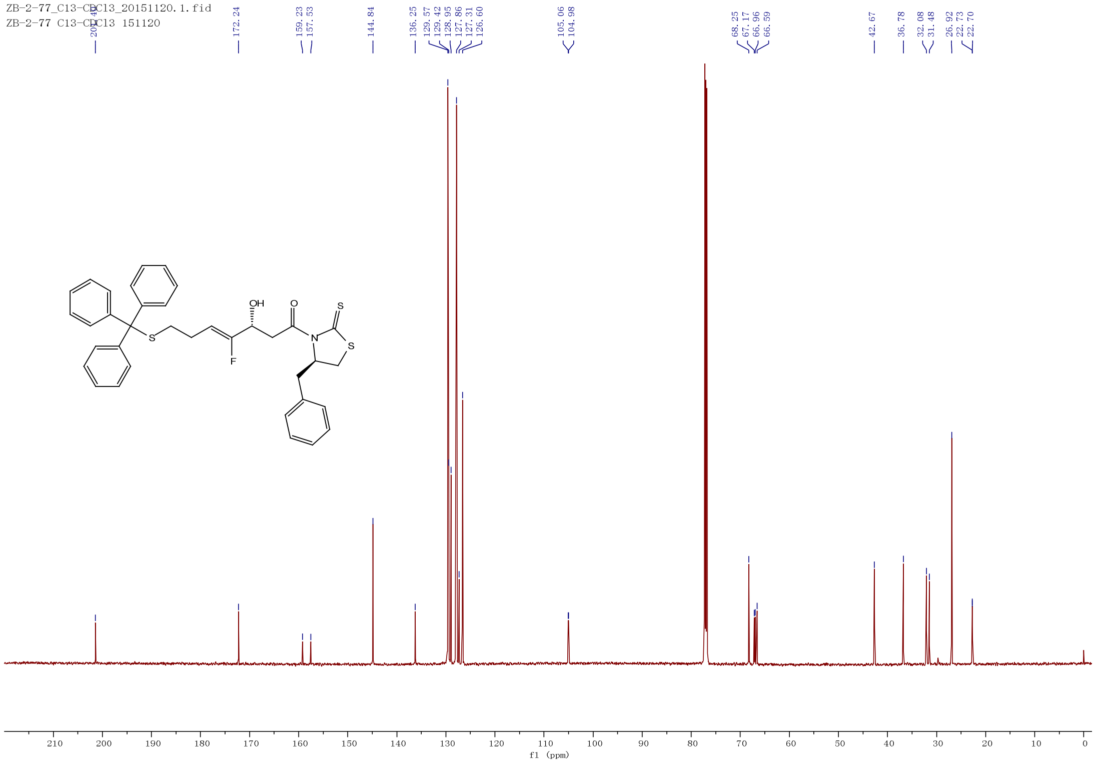

^{19}F NMR Spectra of **9**

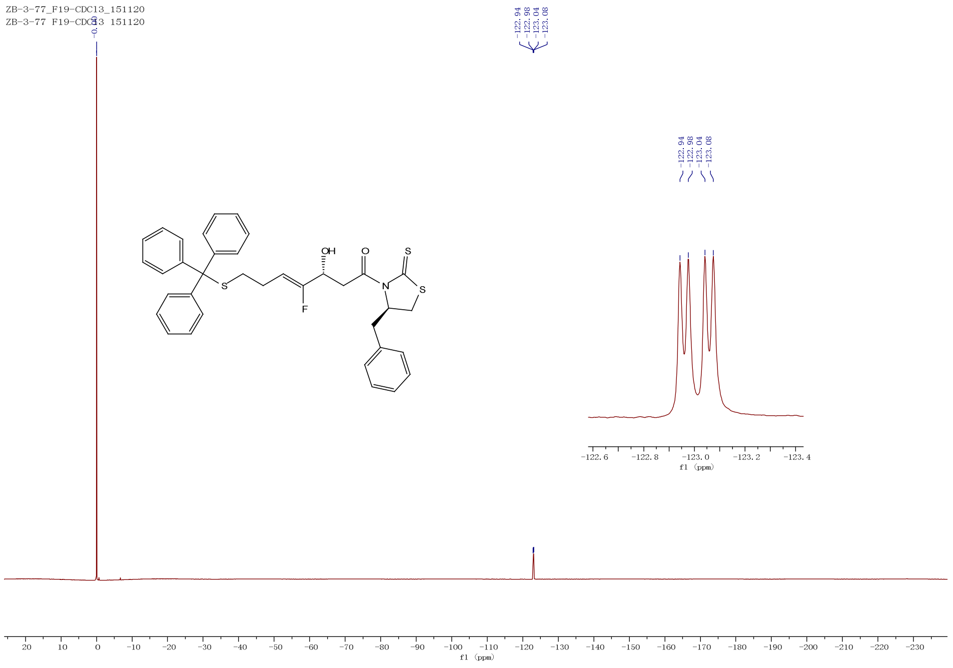

(R)-methyl 2'-(((S,Z)-4-fluoro-3-hydroxy-7-(tritylthio)hept-4-enamido)methyl)-4-methyl-4,5-dihydro-[2,4'-bithiazole]-4-carboxylate (11)

^{1}H NMR Spectra of **11**

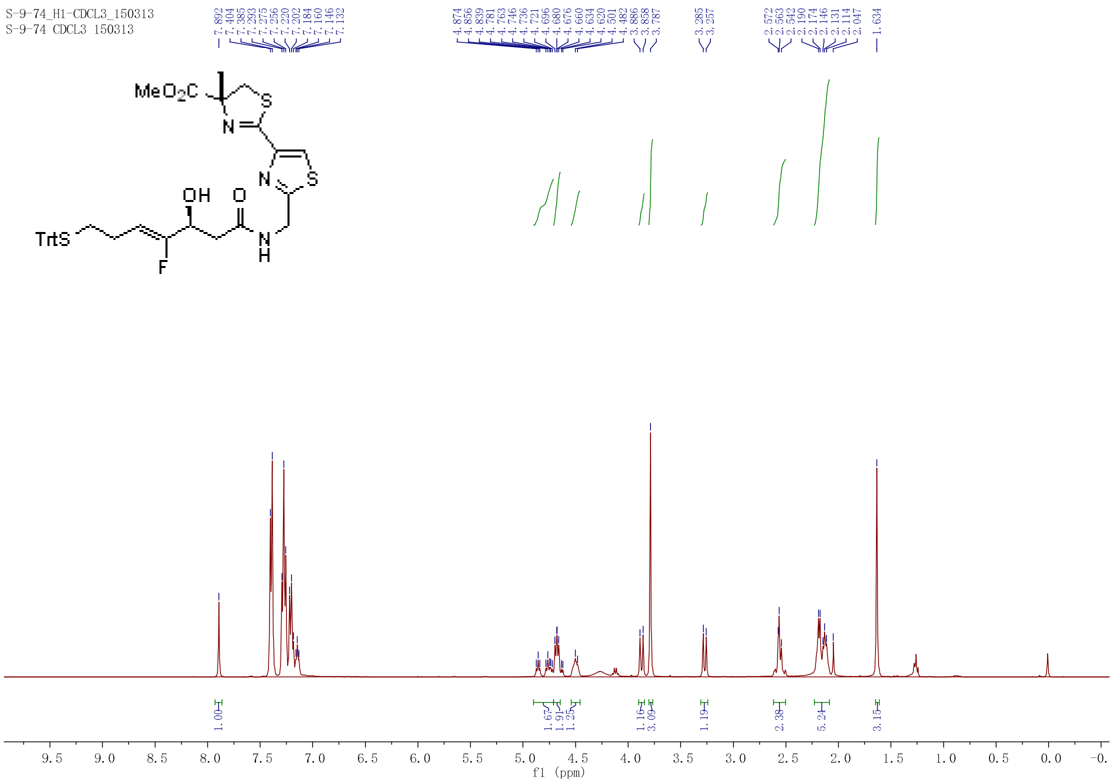

^{13}C NMR Spectra of **11**

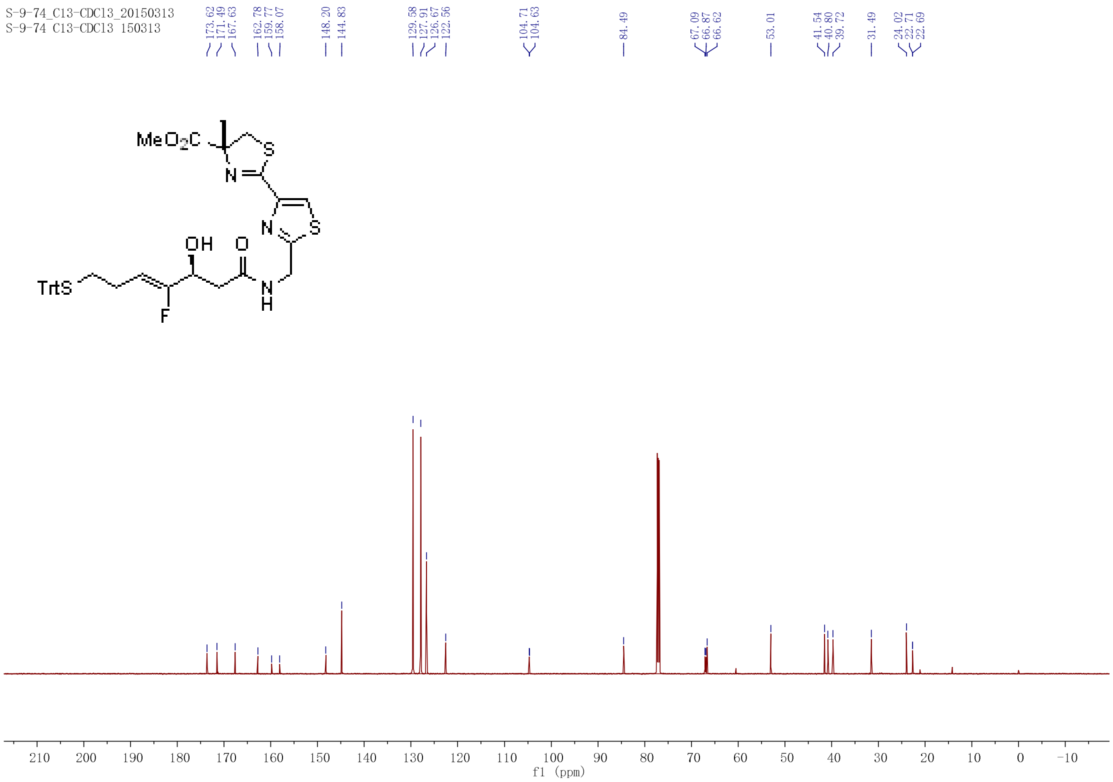

^{19}F NMR Spectra of **11**

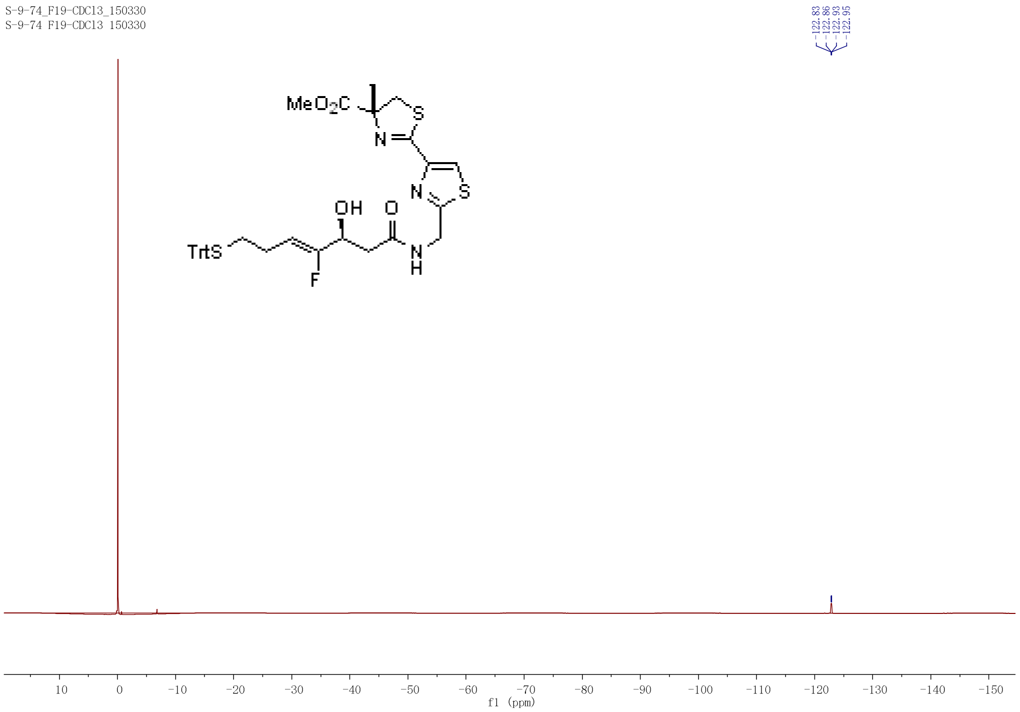

(R)-methyl 2'-((5S,8S)-1-(9H-fluoren-9-yl)-8-((Z)-1-fluoro-4-(tritylthio)but-1-en-1-yl)-5-isopropyl-3,6,10-trioxo-2,7-dioxa-4,11-diazadodecan-12-yl)-4-methyl-4,5-dihydro-[2,4'-bithiazole]-4-carboxylate (13a).

^{1}H NMR Spectra of **13a**

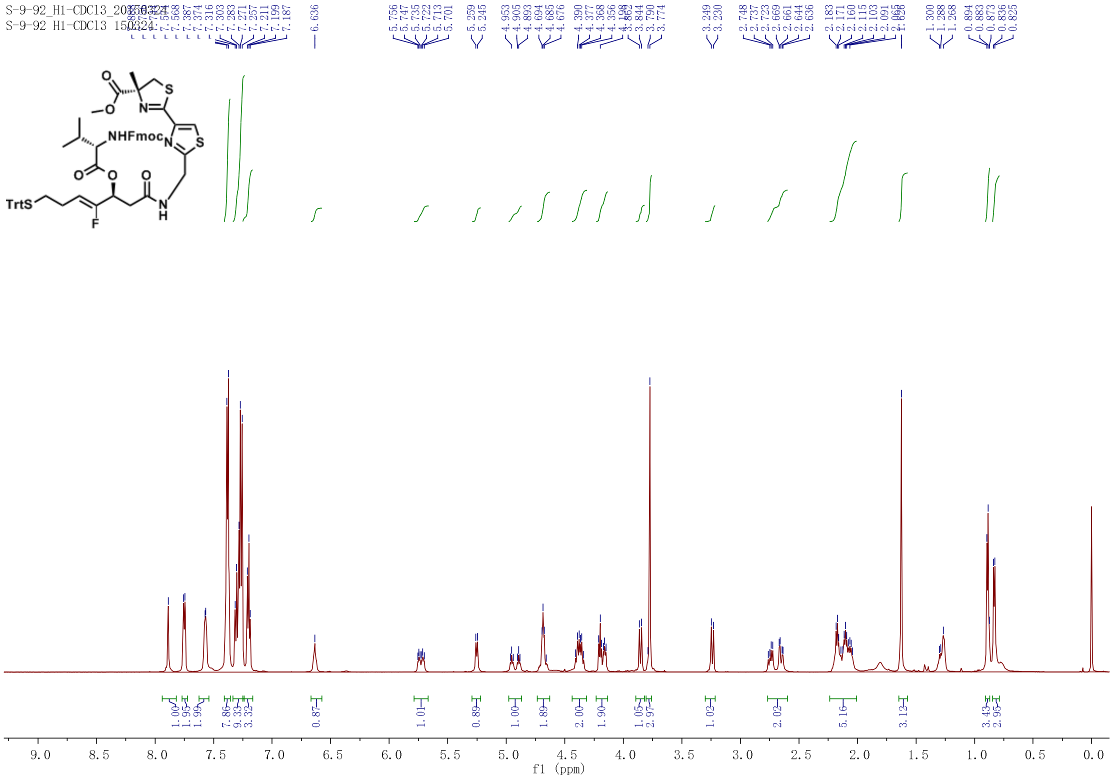

^{13}C NMR Spectra of **13a**

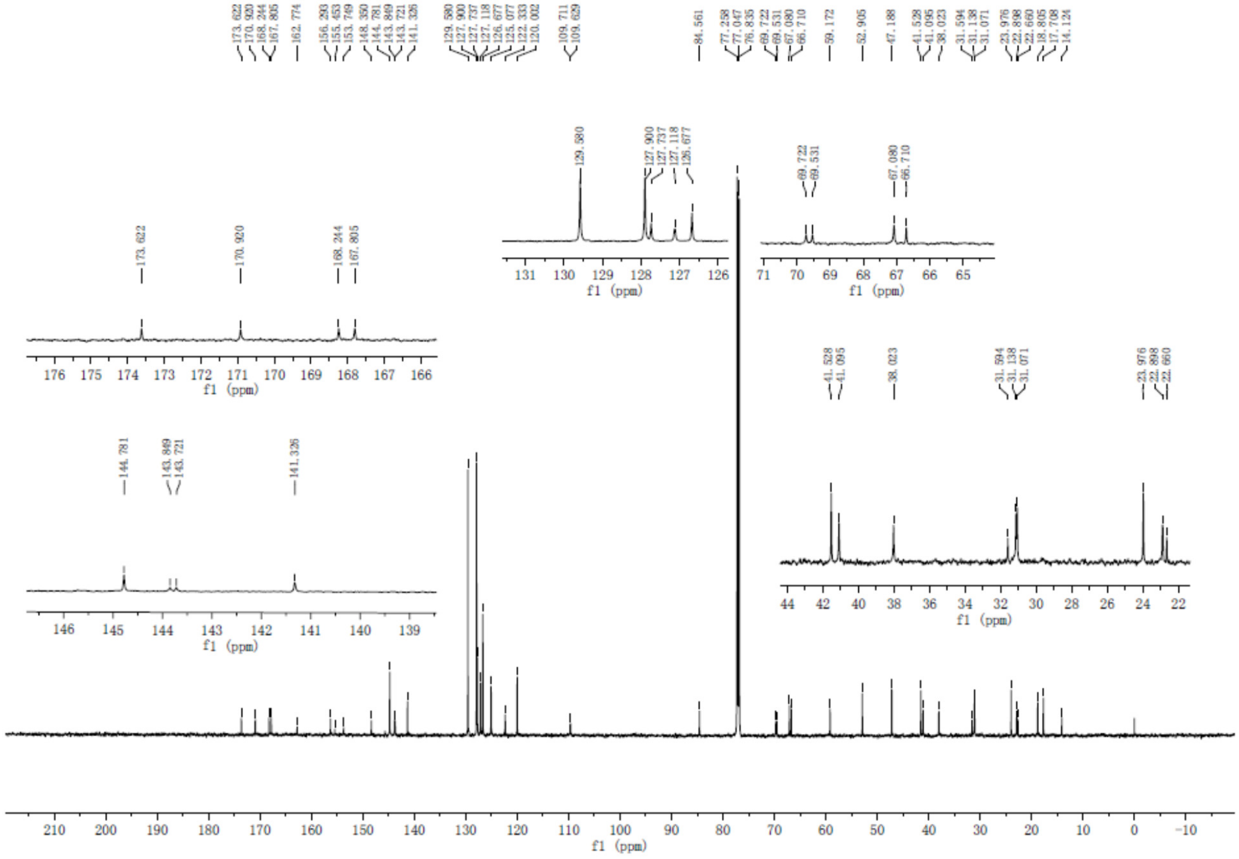

^{19}F NMR Spectra of **13a**

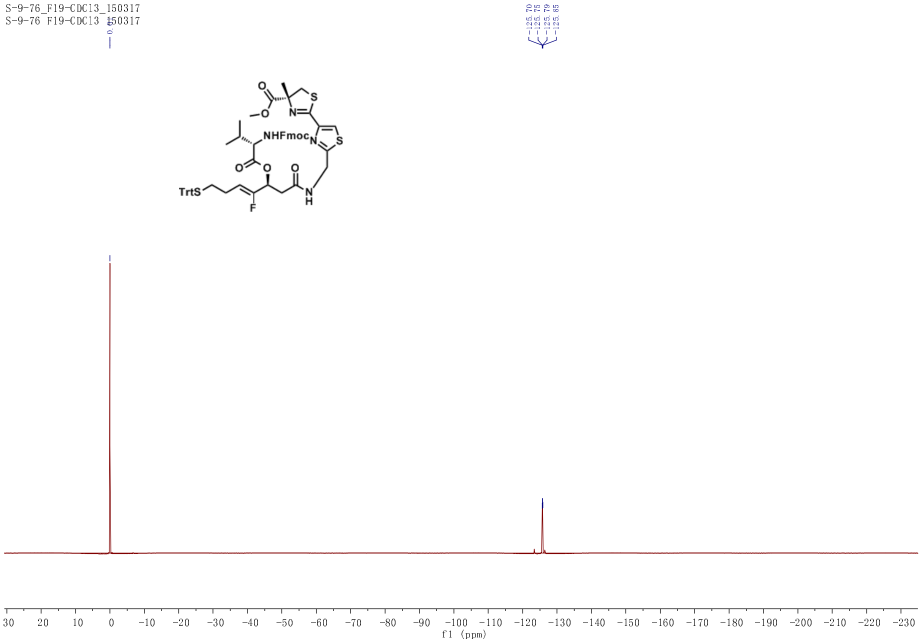

H-H COSY Spectra of**13a**

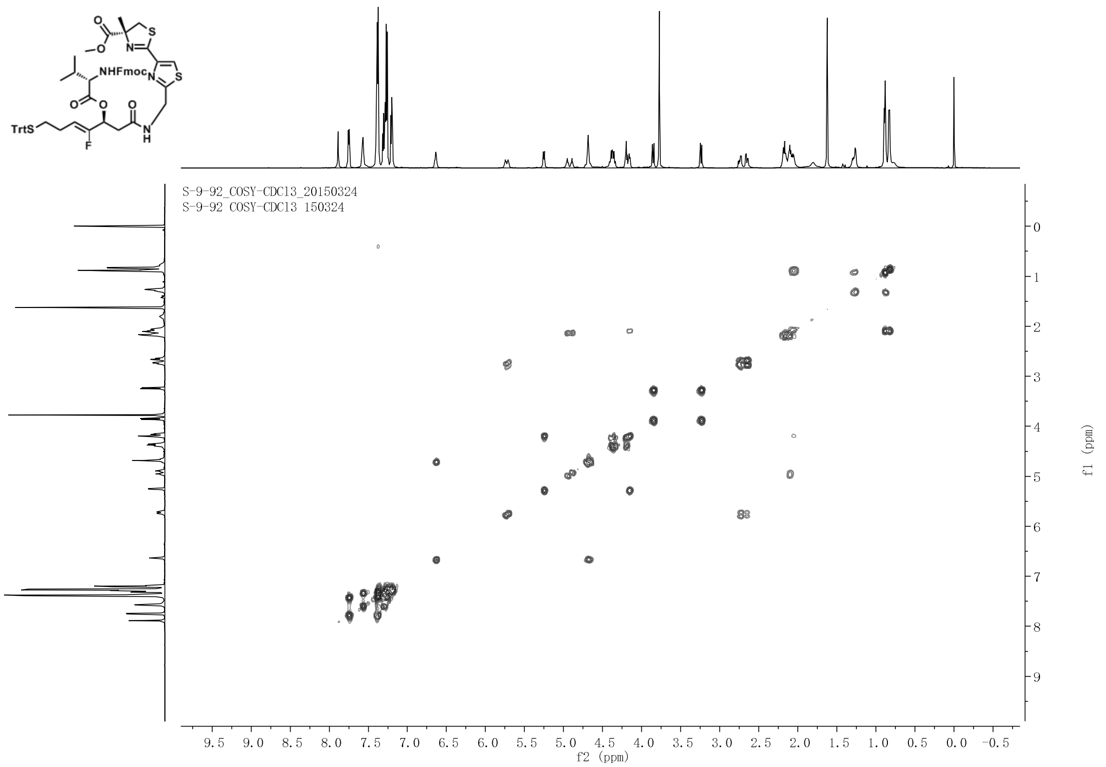

H-C HMQC Spectra of**13a**

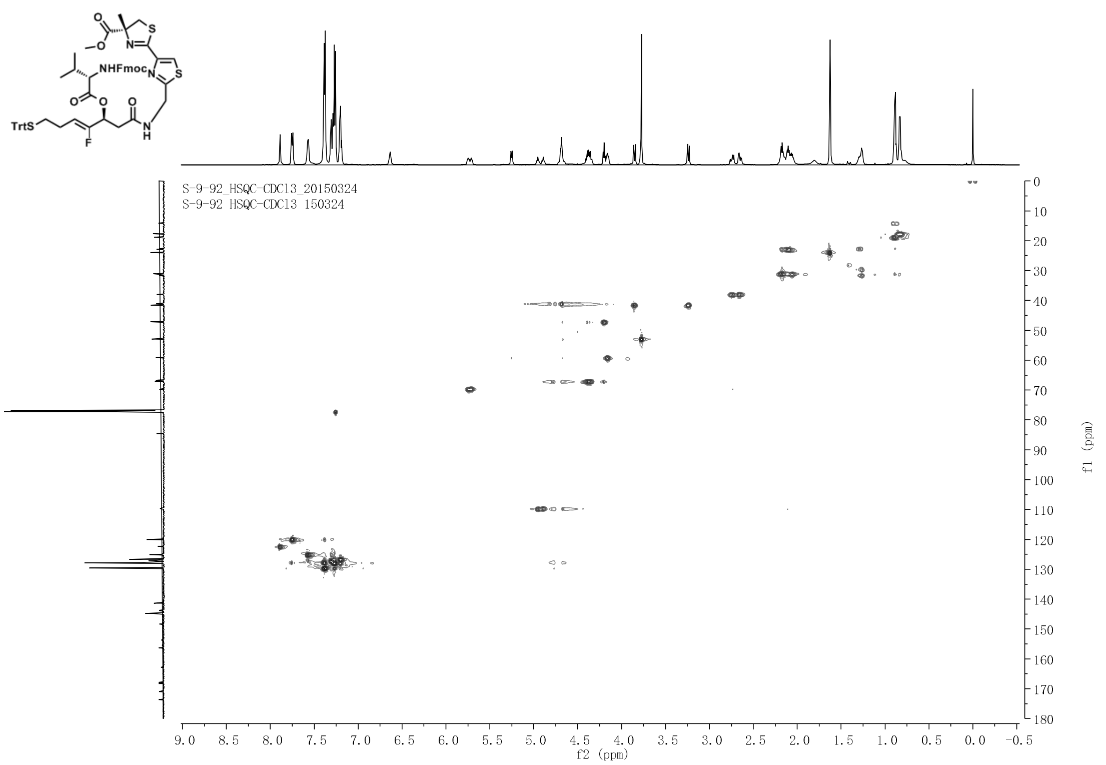

H-C HMBC Spectra of **13a**

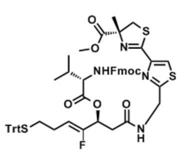

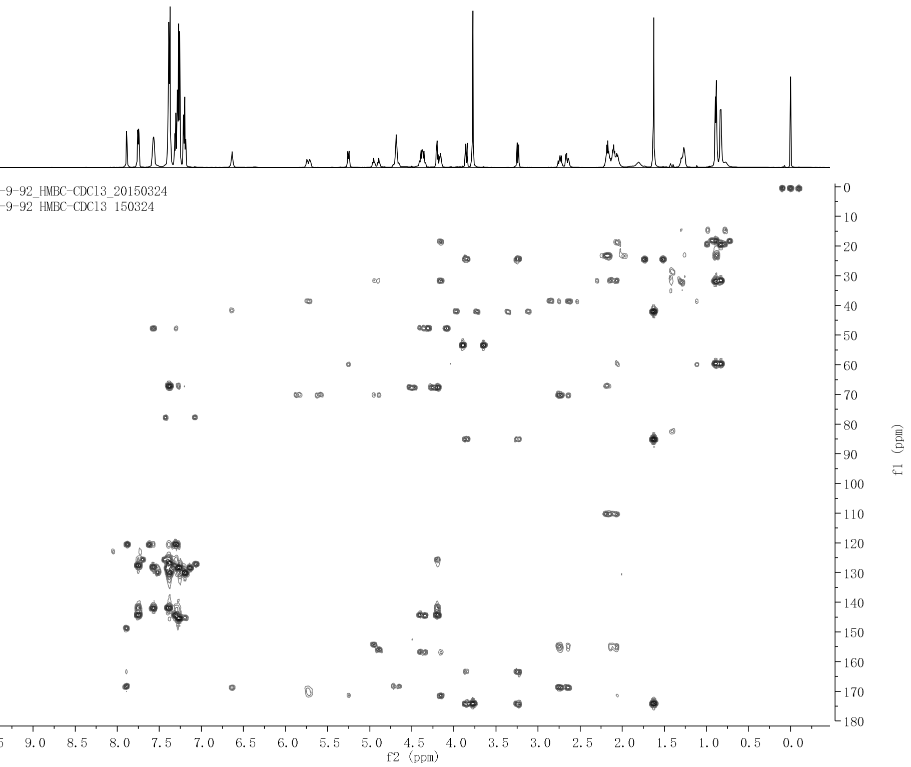

(4R)-methyl 2'-((5S)-5-benzyl-1-(9H-fluoren-9-yl)-8-((Z)-1-fluoro-4-(tritylthio)but-1-en-1-yl)-3,6,10-trioxo-2,7-dioxa-4,11-diazadodecan-12-yl)-4-methyl-4,5-dihydro-[2,4'-bithiazole]-4-carboxylate (13b)

^{1}H NMR Spectra of **13b**

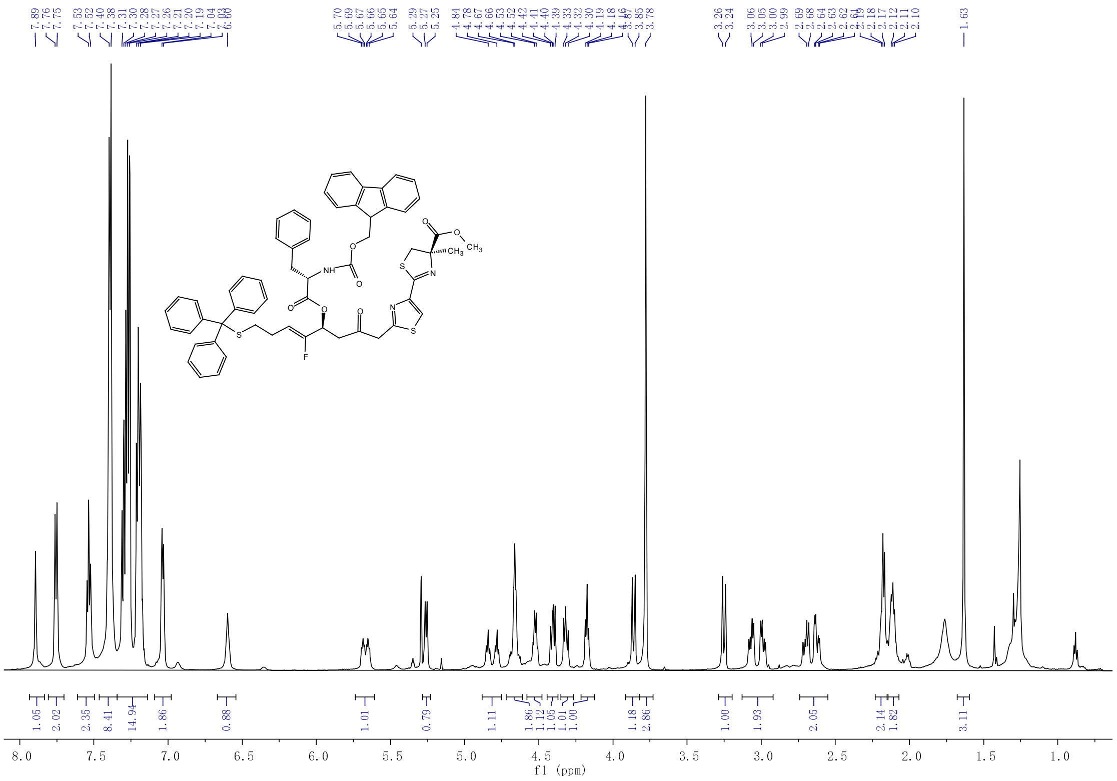

^{13}C NMR Spectra of **13b**

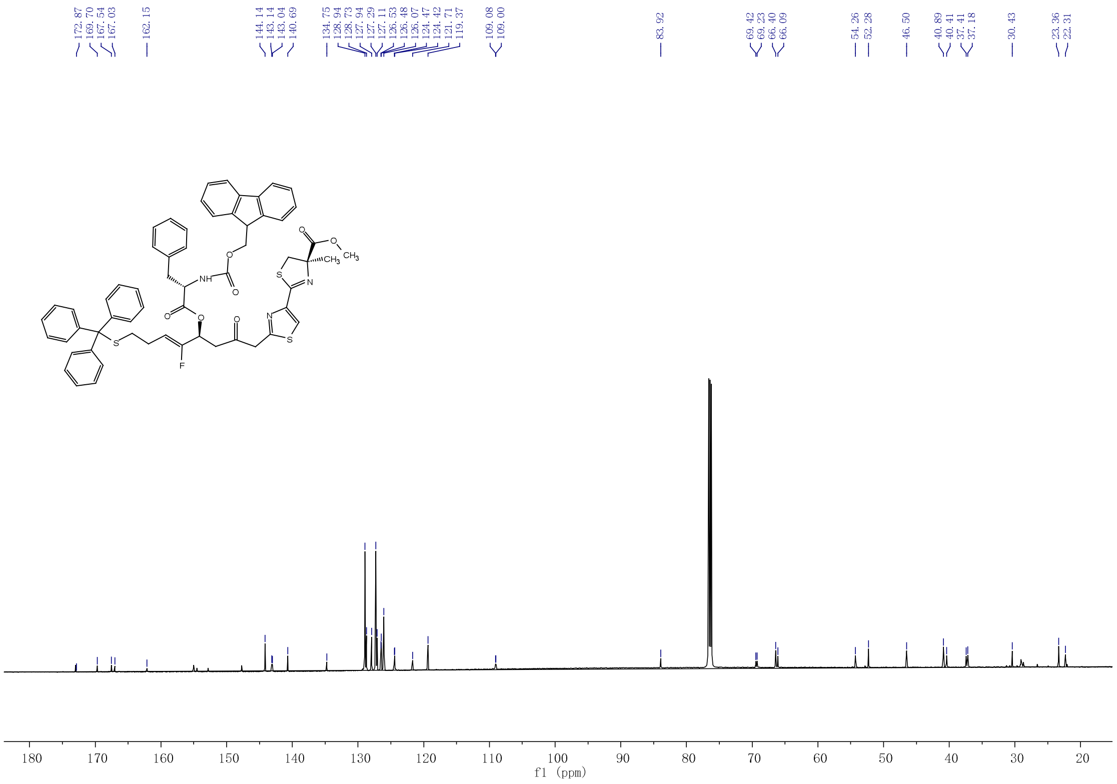

^{19}F NMR Spectra of **13b**

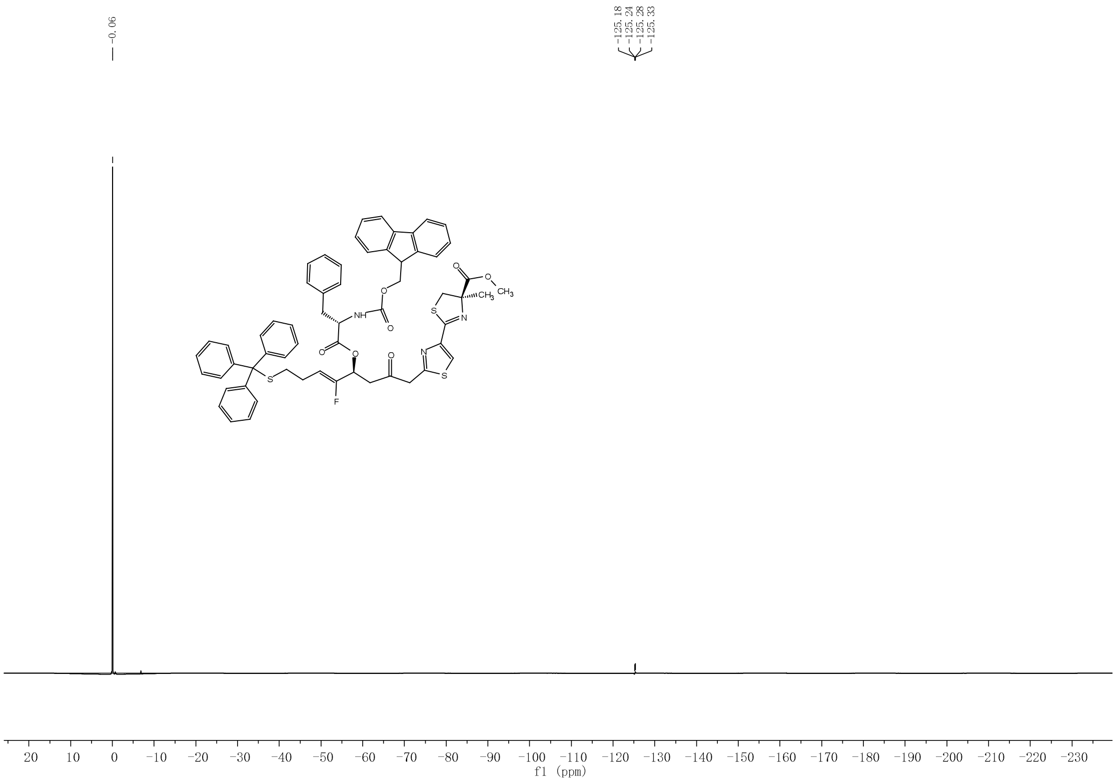

H-H COSY Spectra of**13b**

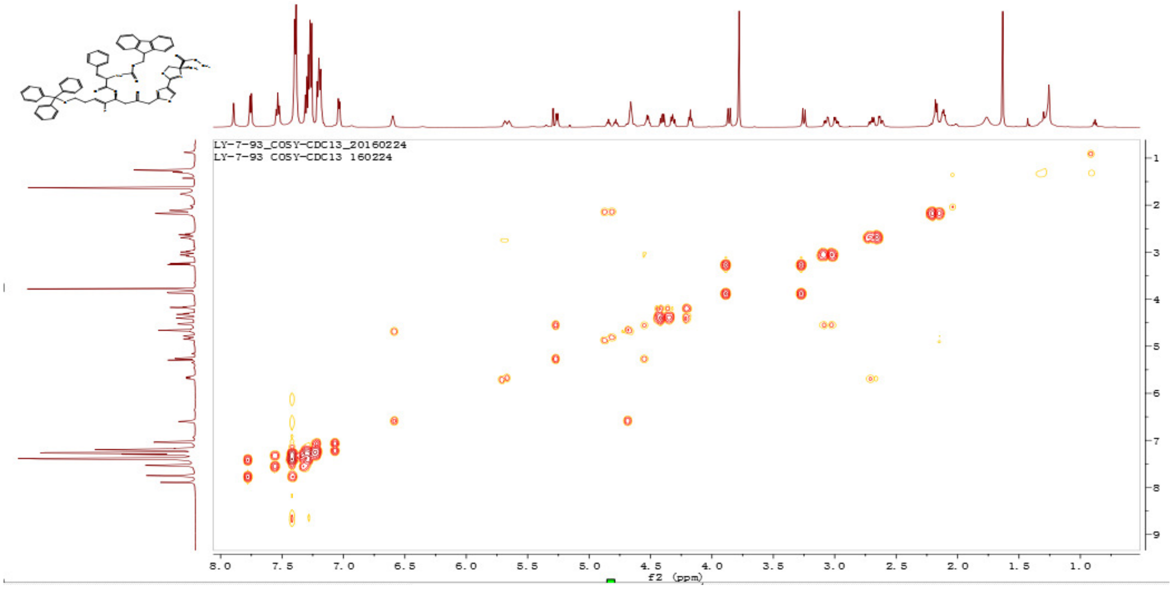

H-C HMQC Spectra of**13b**

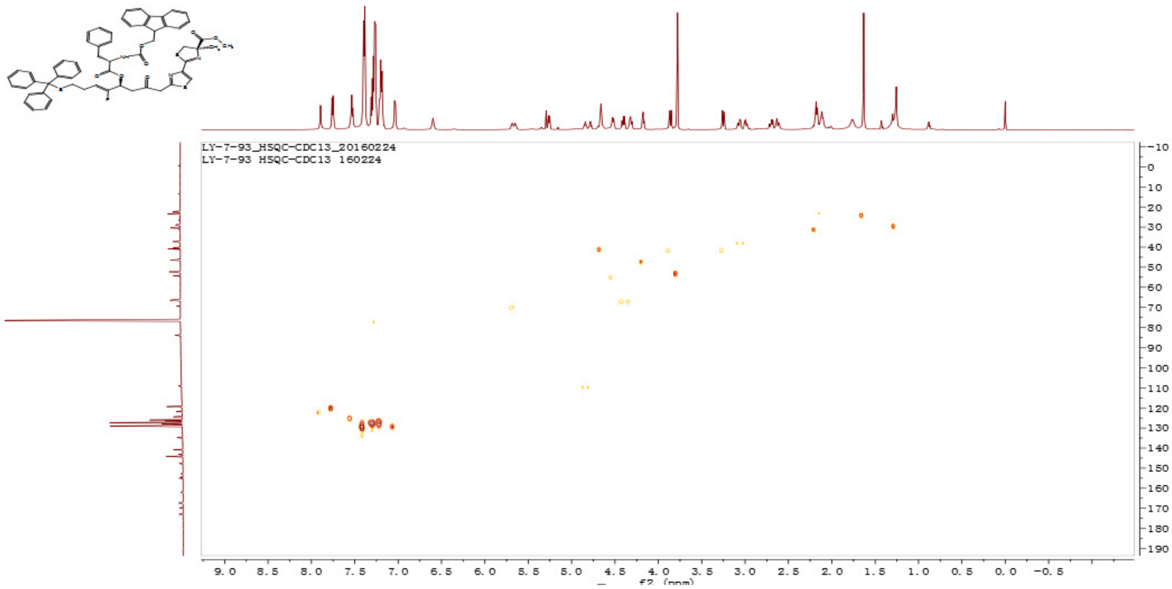

H-C HMBC Spectra of**13b**

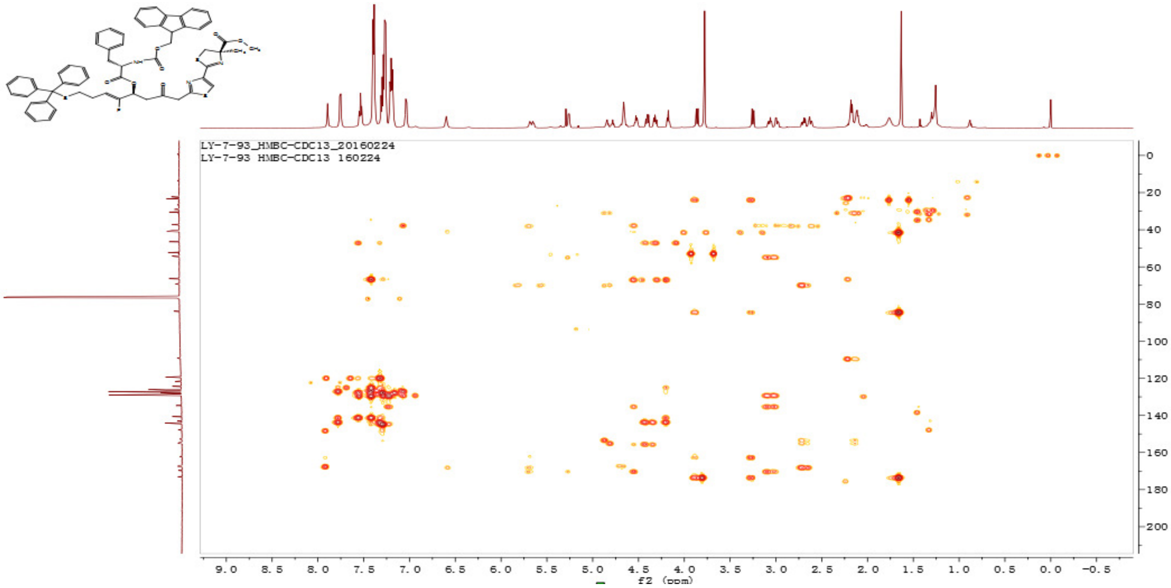

(5R,8S,11S)-11-((Z)-1-fluoro-4-(tritylthio)but-1-en-1-yl)-8-isopropyl-5-methyl-10-oxa-3,17-dithia-7,14,19,20-tetraazatricyclo[14.2.1.12,5]icosa-1(18),2(20),16(19)-triene-6,9,13-trione (14a).

^{1}H NMR Spectra of**14a**

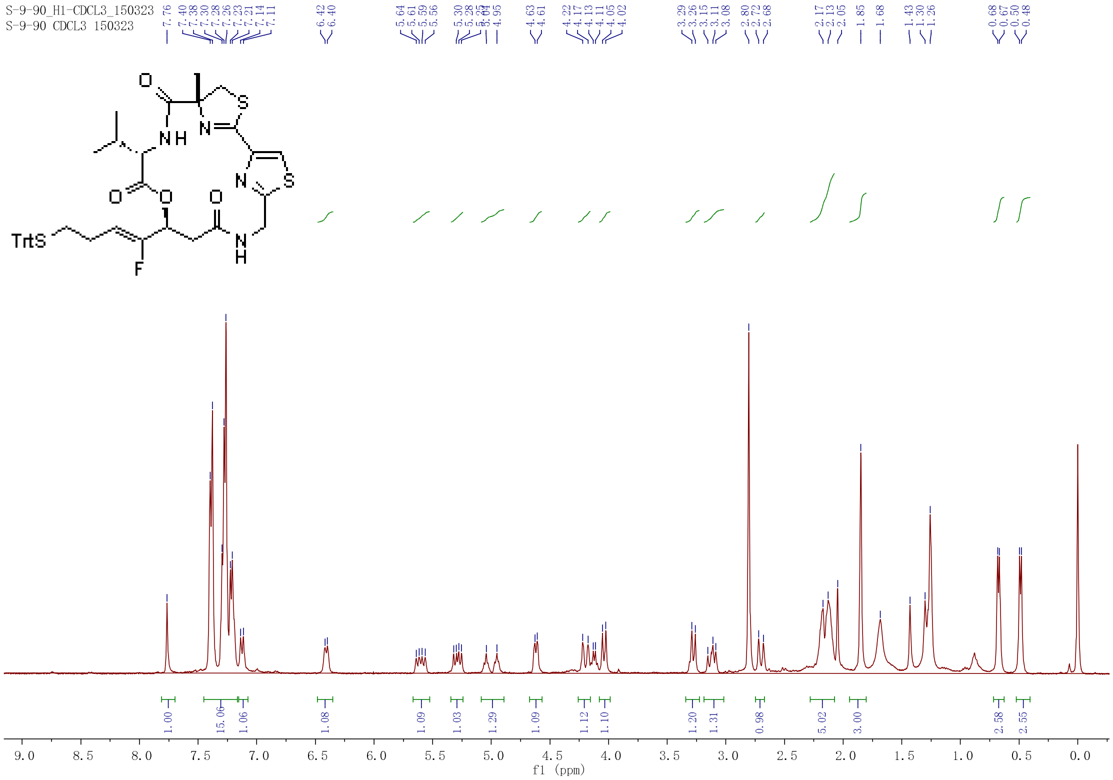

^{13}C NMR Spectra of **14a**

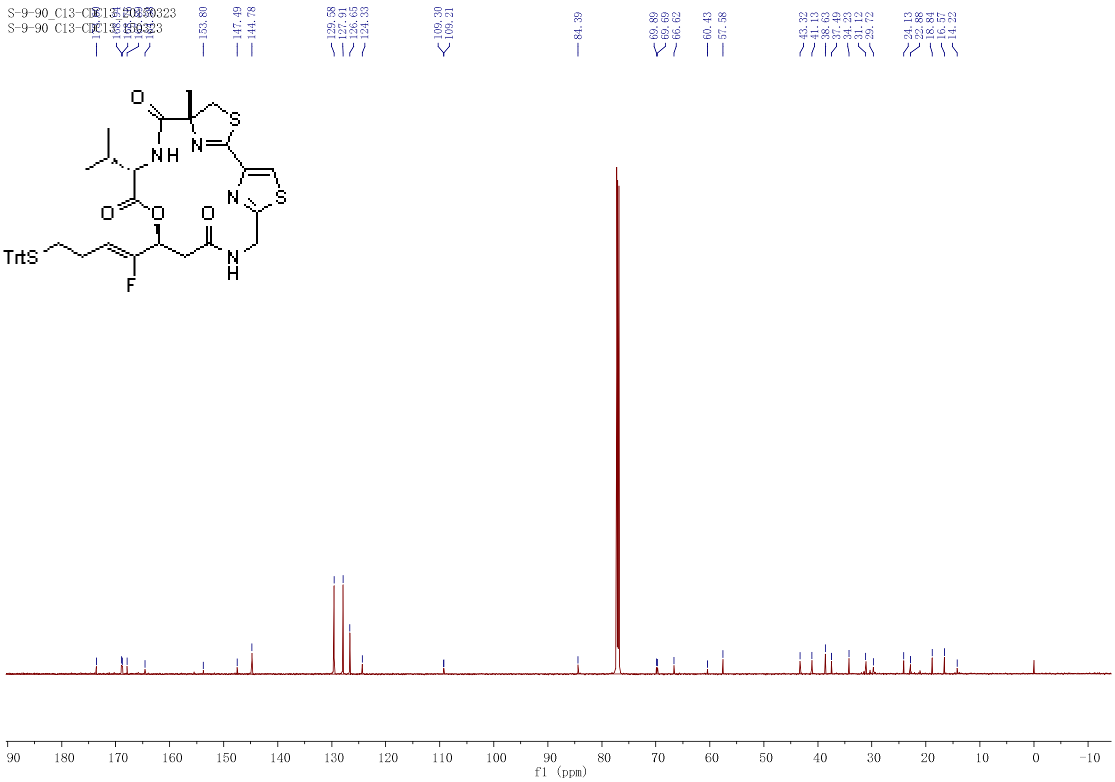

^{19}F NMR Spectra of **14a**

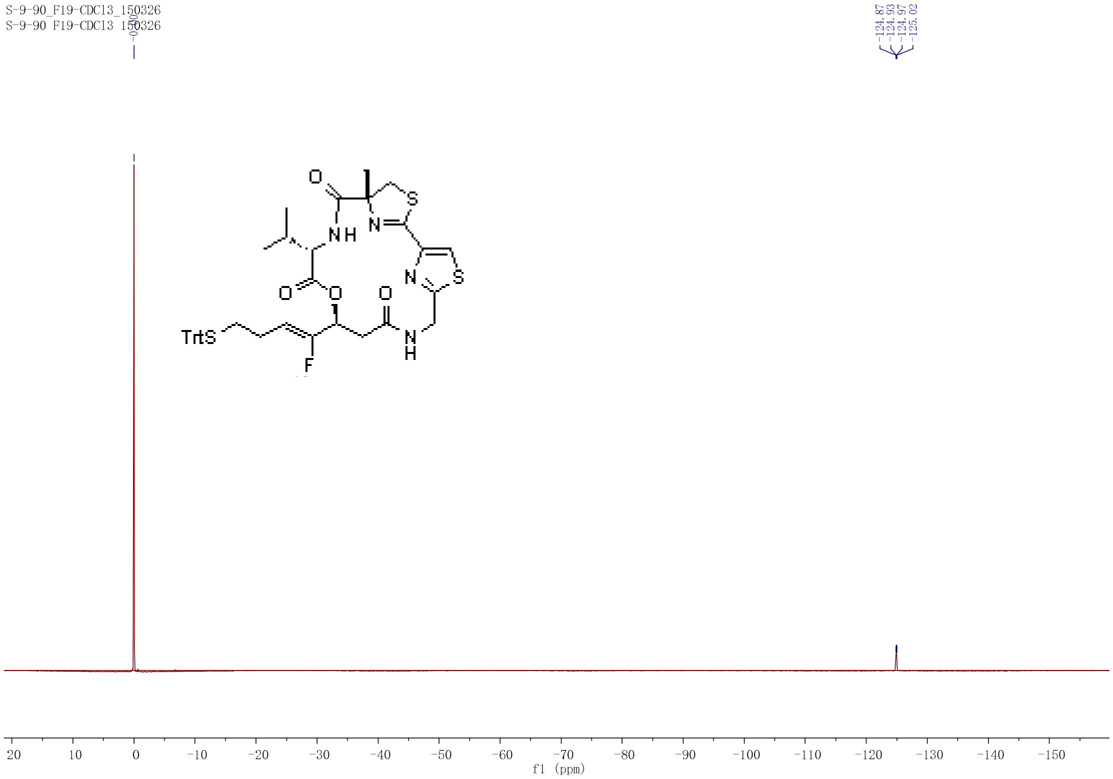

(5R,8S)-8-benzyl-11-((Z)-1-fluoro-4-(tritylthio)but-1-en-1-yl)-5-methyl-10-oxa-3,17-dithia-7,14,19,20-tetraazatricyclo[14.2.1.1^{2,5}]icosa-1(18),2(20),16(19)-triene-6,9,13-trione (14b)

^{1}H NMR Spectra of**14b**

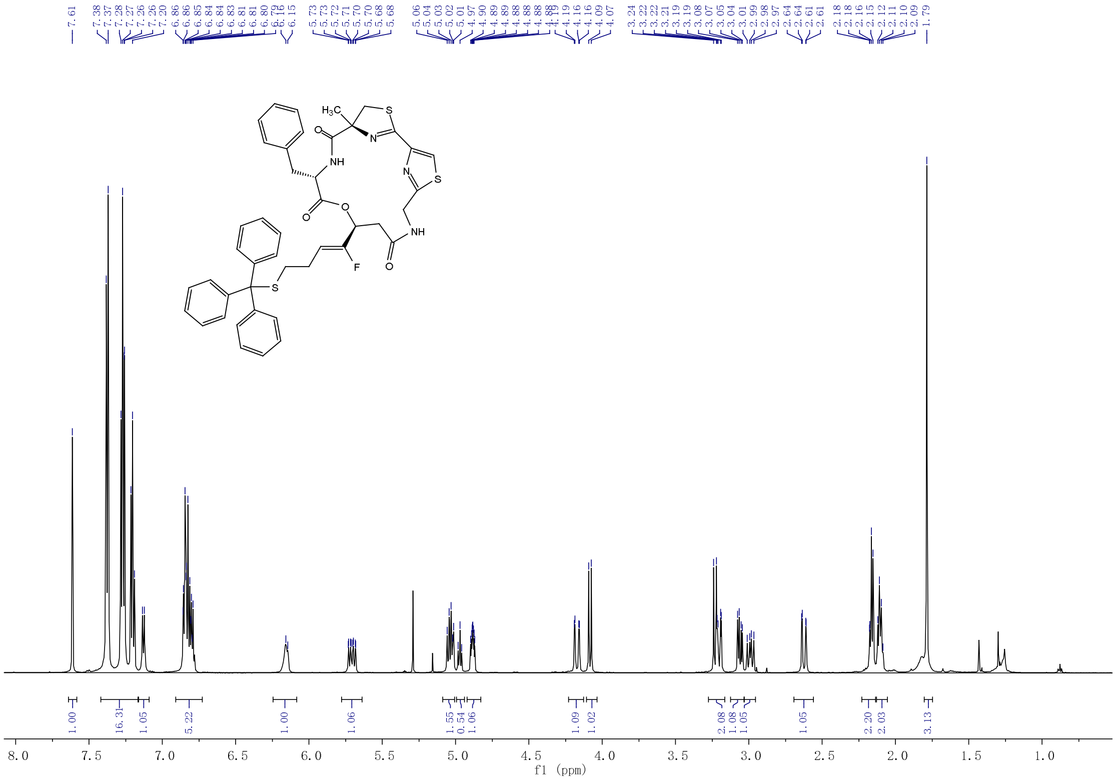

^{13}C NMR Spectra of**14b**

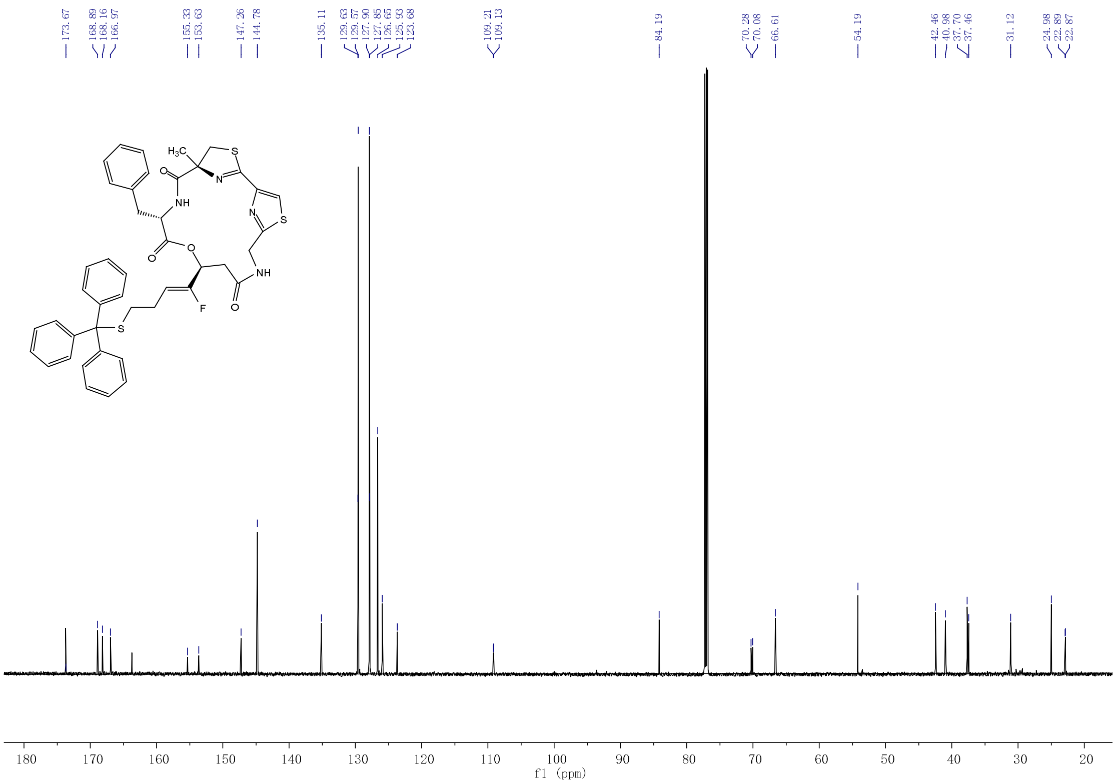

^{19}F NMR Spectra of**14b**

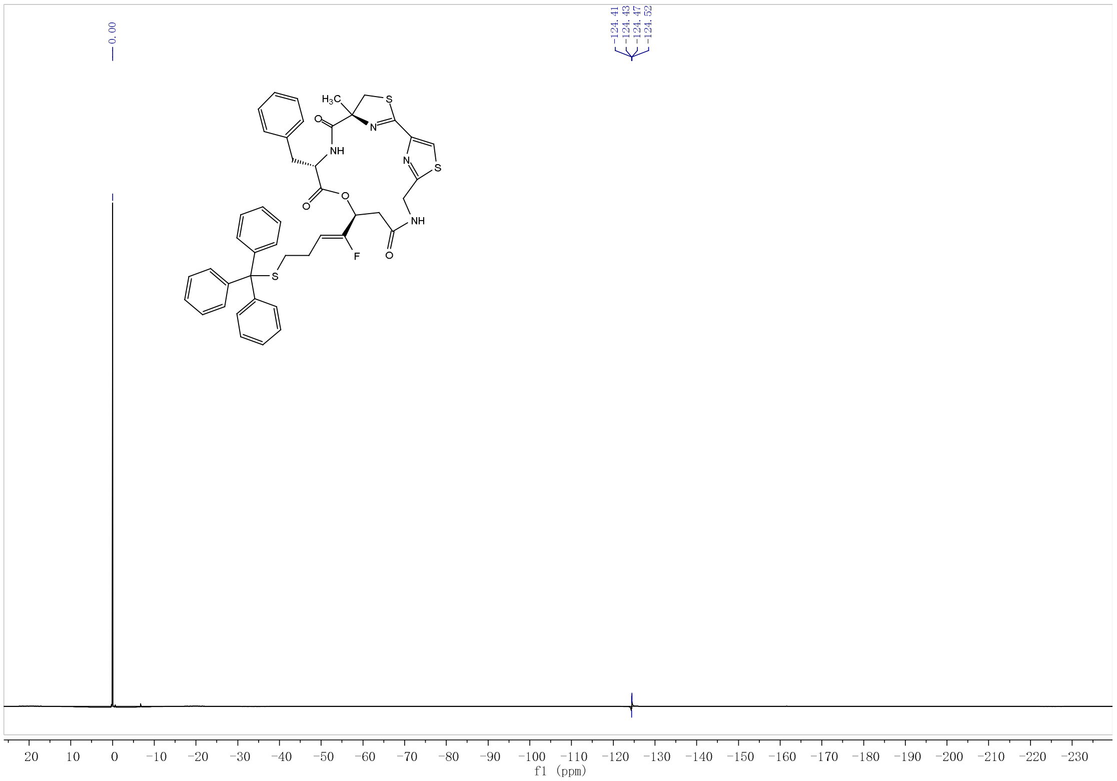

(5R,8S,11S)-11-((Z)-1-fluoro-4-mercaptobut-1-en-1-yl)-8-isopropyl-5-methyl-10-oxa-3,17-dithia-7,14,19,20-tetraazatricyclo[14.2.1.1^{2,5}]icosa-1(18),2(20),16(19)-triene-6,9,13-trione (15a).

^{1}H NMR Spectra of**15a**

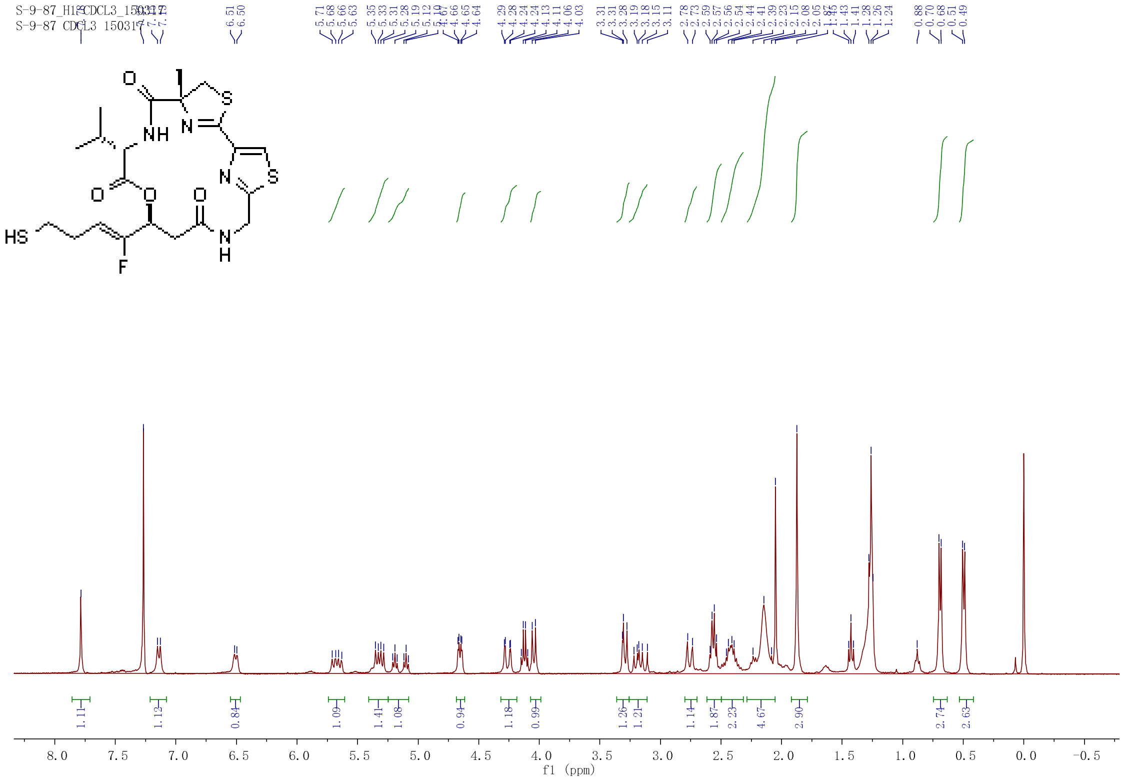

(5R,8S)-8-benzyl-11-((Z)-1-fluoro-4-mercaptobut-1-en-1-yl)-5-methyl-10-oxa-3,17-dithia-7,14,19,20-tetraazatricyclo[14.2.1.1^{2,5}]icosa-1(18),2(20),16(19)-triene-6,9,13-trione (15b)

^{1}H NMR Spectra of**15b**

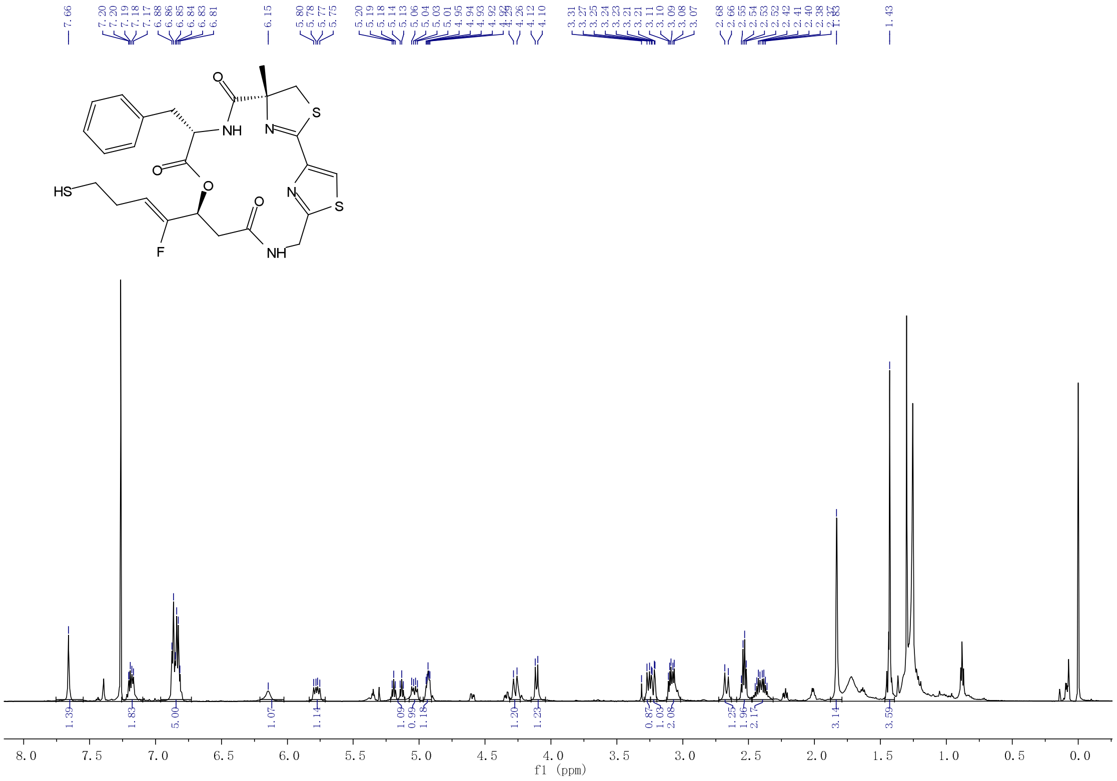

^{19}F NMR Spectra of**15b**

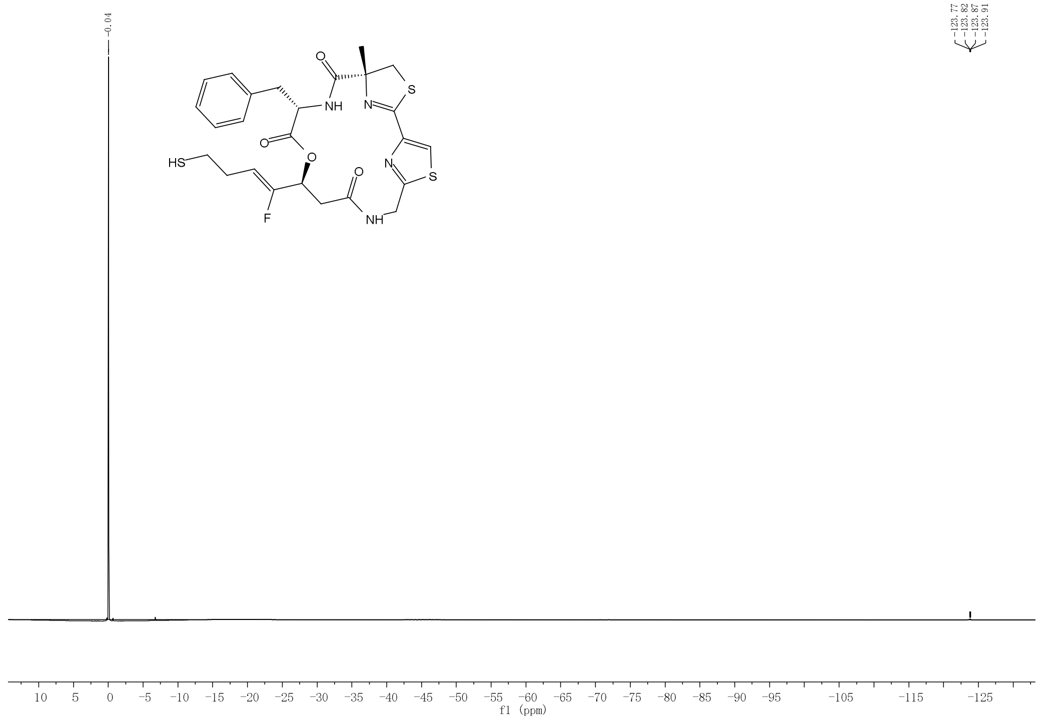

S-((Z)-4-fluoro-4-((5R,8S,11S)-8-isopropyl-5-methyl-6,9,13-trioxo-10-oxa-3,17-dithia-7,14,19,20-tetraazatricyclo[14.2.1.12,5]icosa-1(18),2(20),16(19)-trien-11-yl)but-3-en-1-yl) octanethioate (16a).

^{1}H NMR Spectra of **16a**

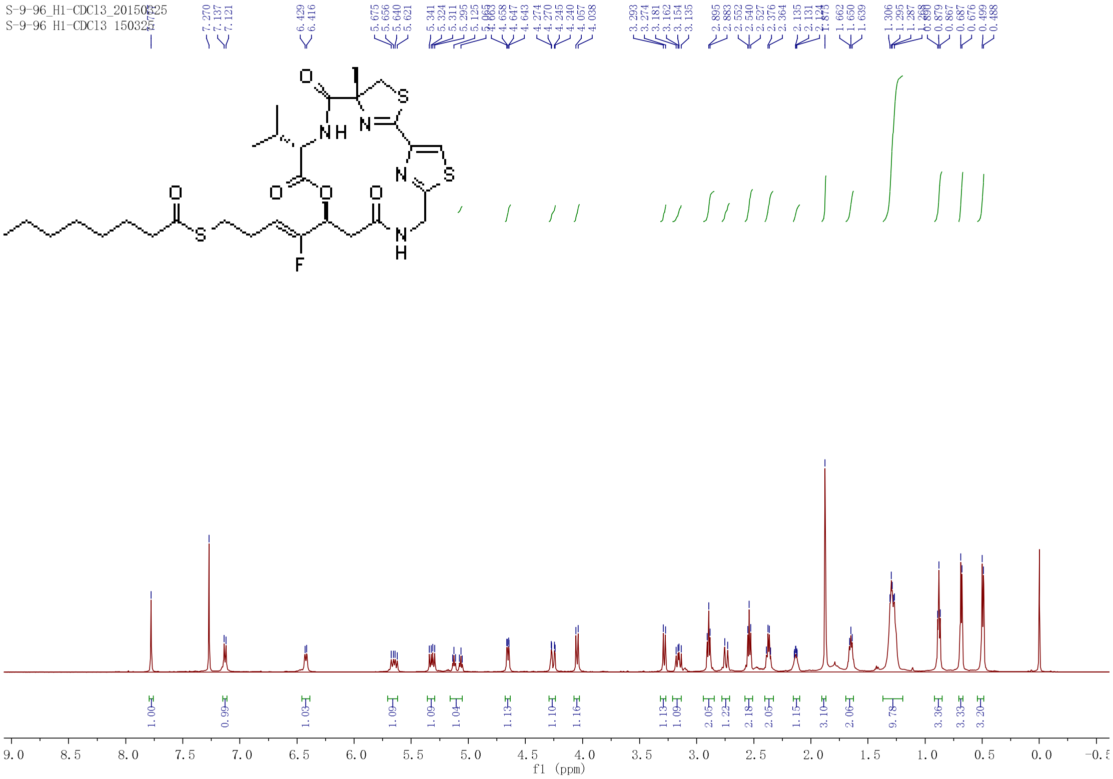

^{13}C NMR Spectra of **16a**

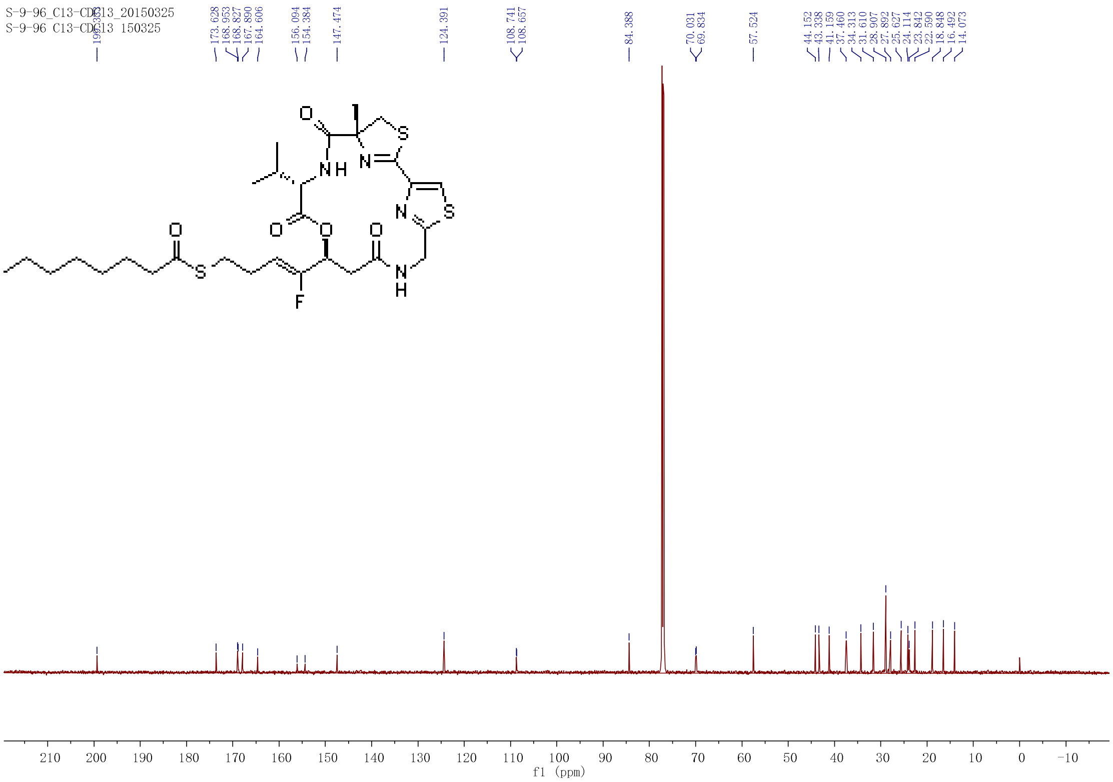

^{19}F NMR Spectra of **16a** (CF_3Cl and CF_3CO_2H as the reference)

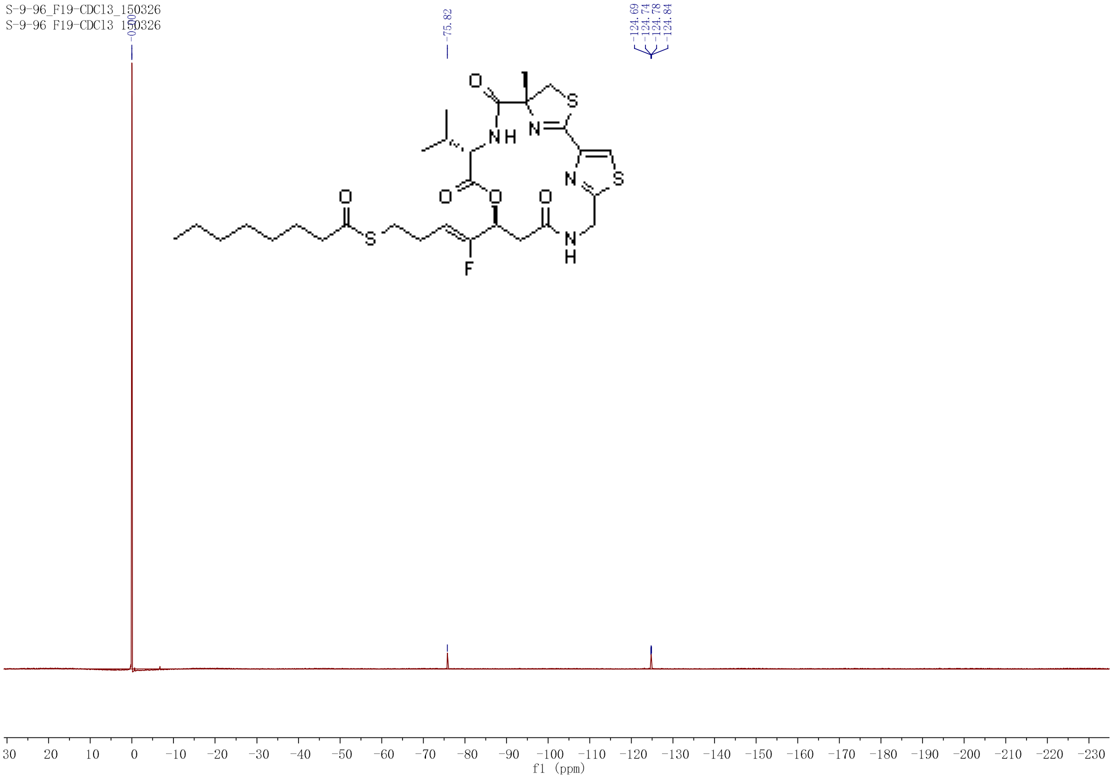

H-H COSY Spectra of **16a**

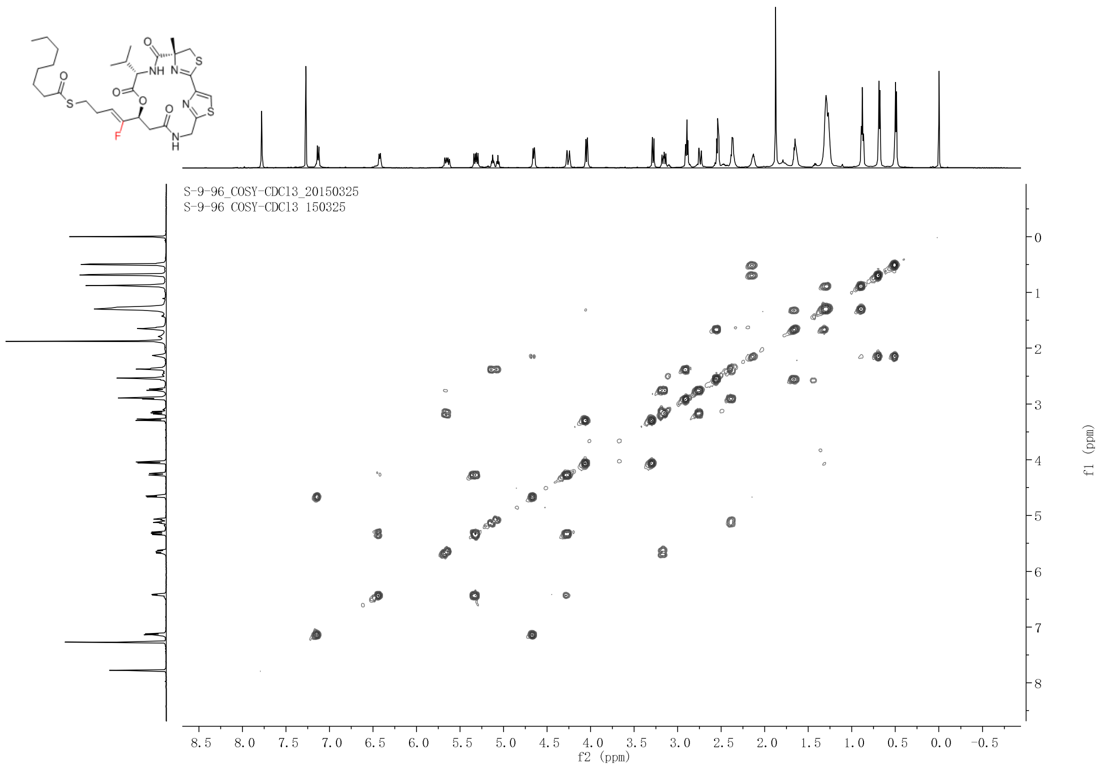

H-C HMQC Spectra of **16a**

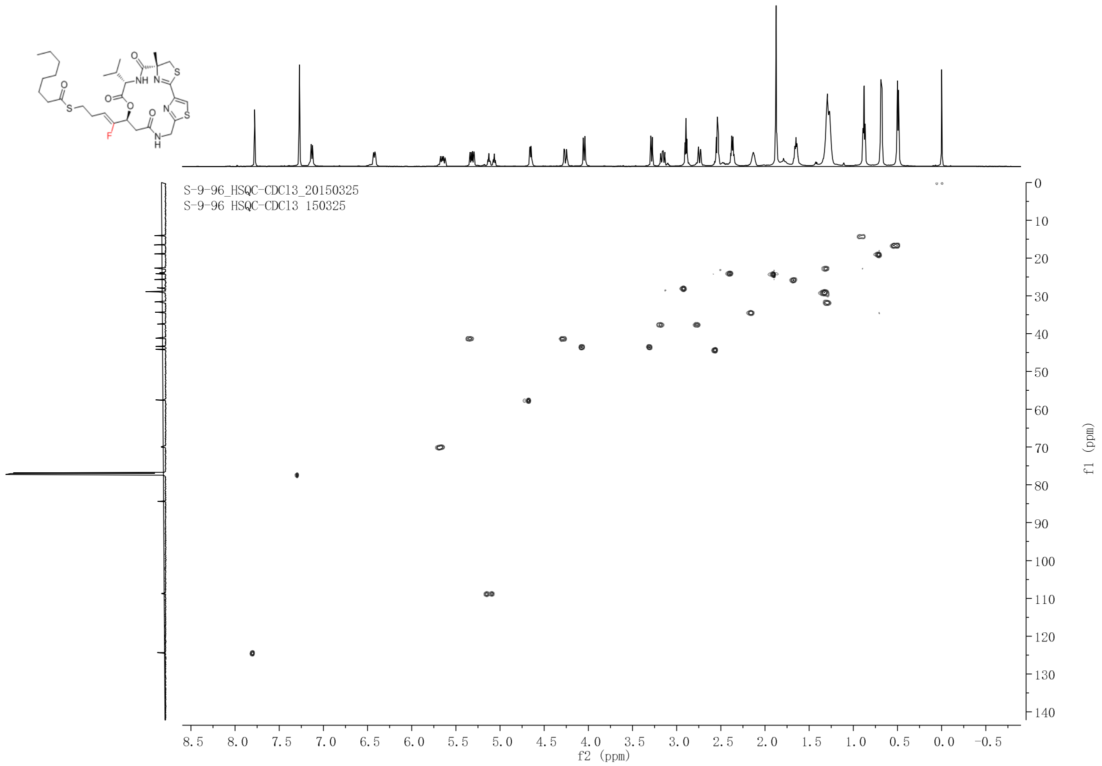

H-C HMBC Spectra of **16a**

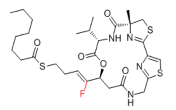

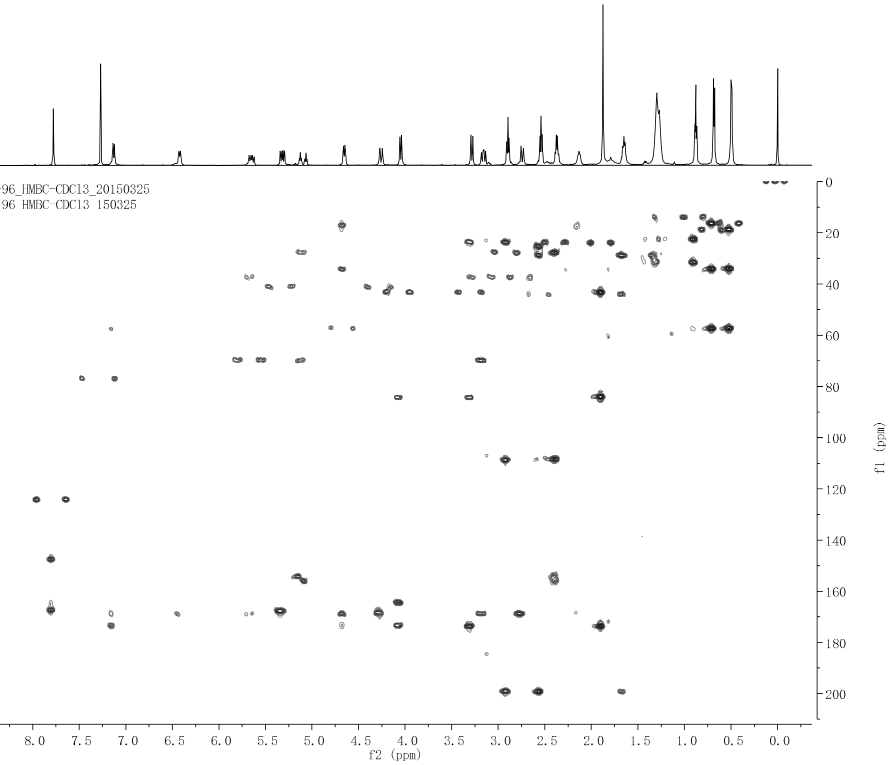

S-((Z)-4-((5R,8S)-8-benzyl-5-methyl-6,9,13-trioxo-10-oxa-3,17-dithia-7,14,19,20-tetraazatricyclo[14.2.1.1^{2,5}]icosa-1(18),2(20),16(19)-trien-11-yl)-4-fluorobut-3-en-1-yl) octanethioate (16b)

^{1}H NMR Spectra of **16b**

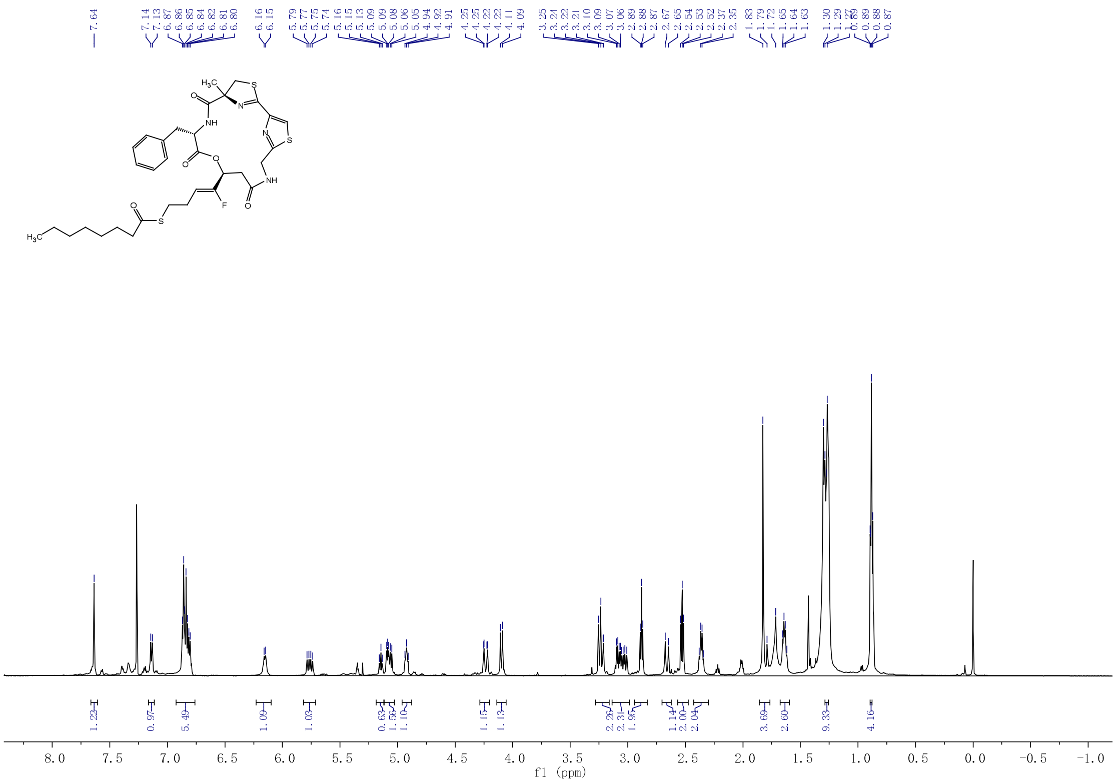

^{13}C NMR Spectra of **16b**

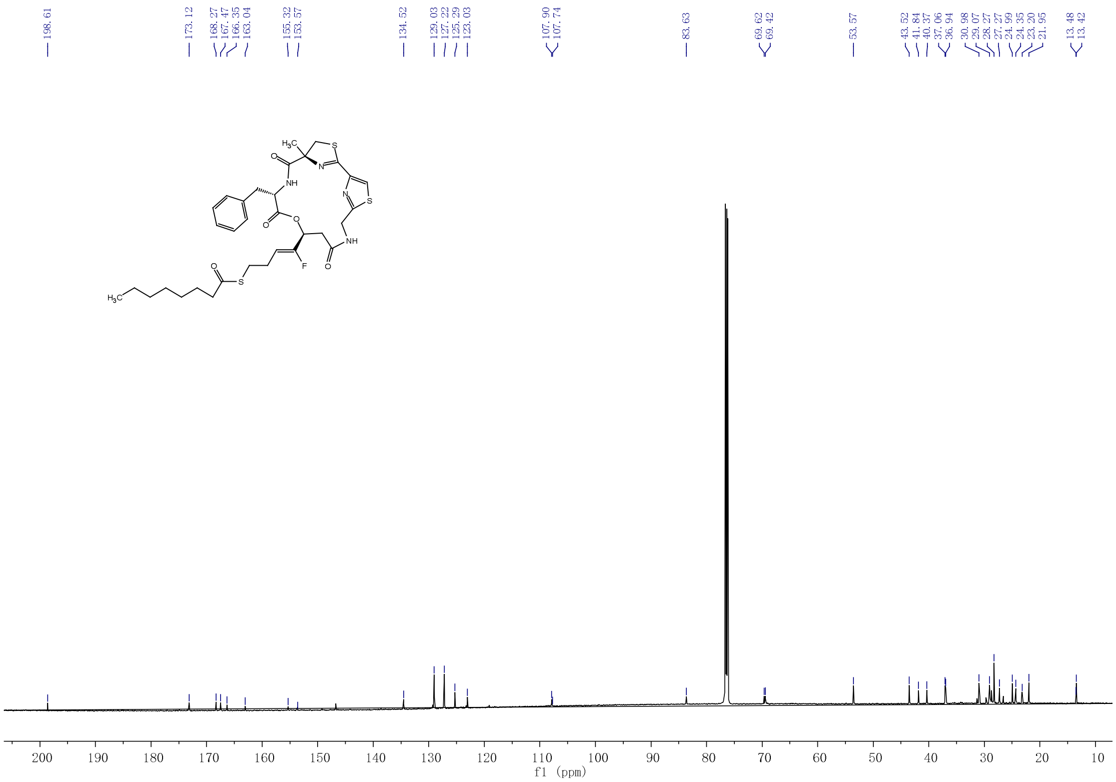

^{19}F NMR Spectra of **16b**

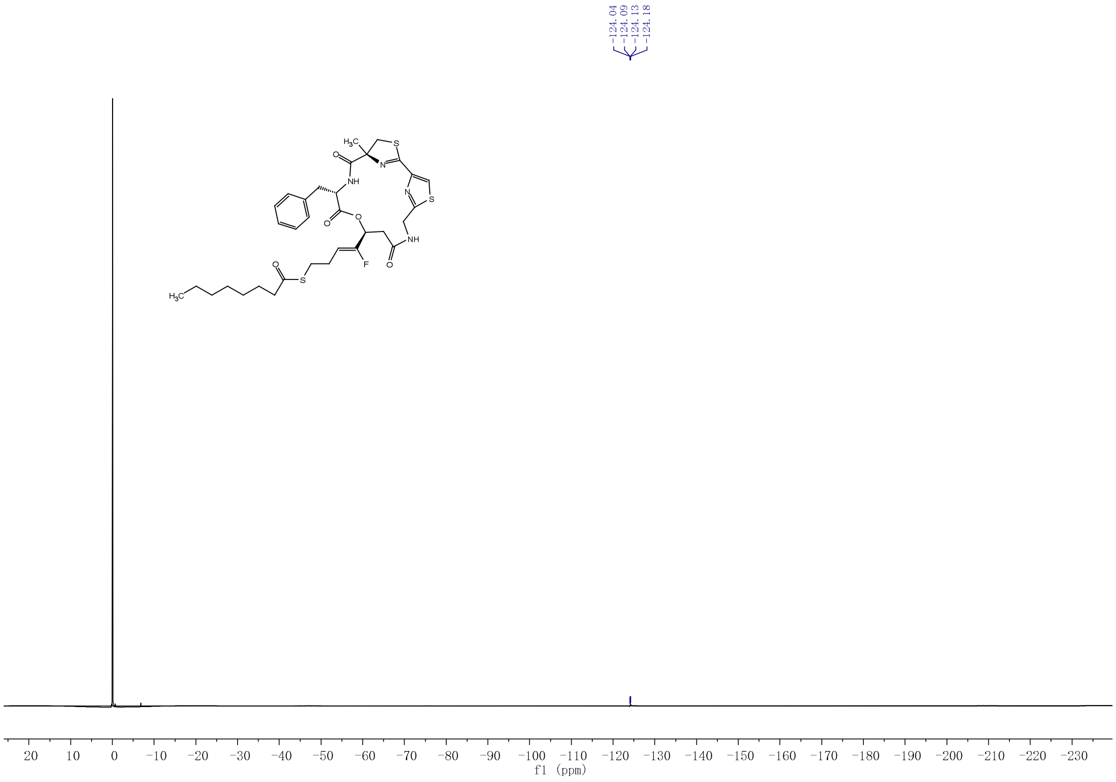

Largazole

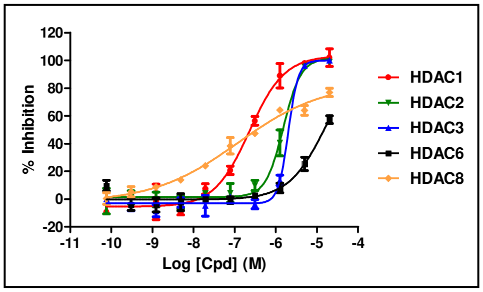

Largazole thiol

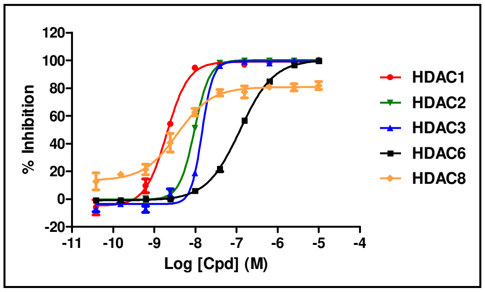

16a

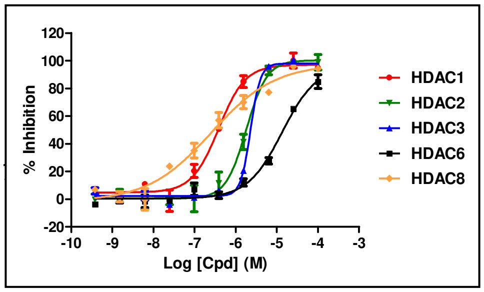

15a

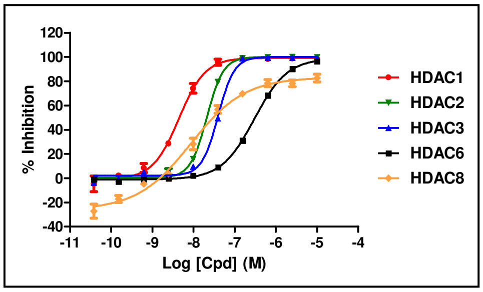

15b

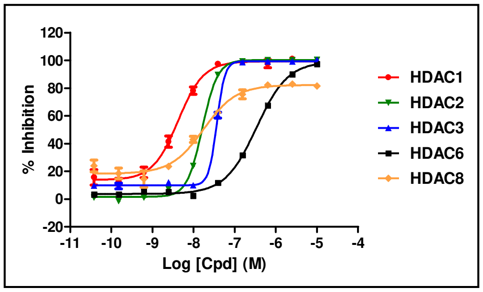

SAHA

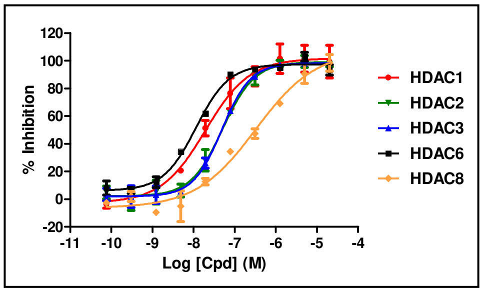

The concentration-response curves of the compounds in the enzymatic assays (HDAC 1, 2, 3, 8 and 6).

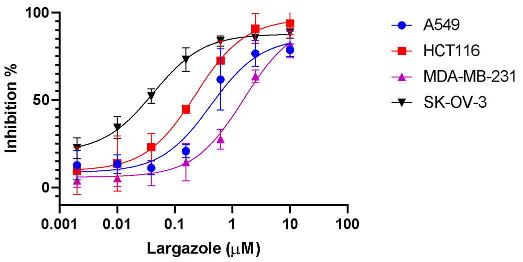

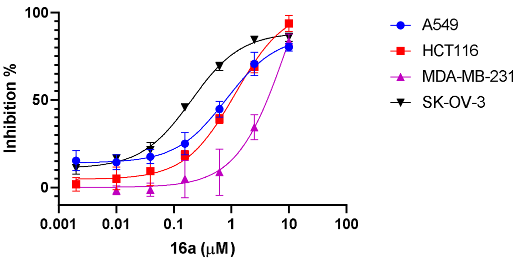

The concentration-response curves of the compounds in the cellular assays
